# Expression of microRNAs and isomiRs in the porcine endometrium: implications for gene regulation at the maternal-conceptus interface

**DOI:** 10.1186/s12864-015-2172-2

**Published:** 2015-11-06

**Authors:** Kamil Krawczynski, Stefan Bauersachs, Zaneta P. Reliszko, Alexander Graf, Monika M. Kaczmarek

**Affiliations:** Institute of Animal Reproduction and Food Research Polish Academy of Sciences (IARFR PAS), Tuwima 10, 10-748 Olsztyn, Poland; Laboratory for Functional Genome Analysis (LAFUGA), LMU University of Munich, Feodor-Lynen-Strasse 25, Munich, 81377 Germany; Present address: Department of Environmental Systems Science, ETH Zurich, Animal Physiology, Institute of Agricultural Sciences, Universitaetstrasse 2, Zurich, 8092 Switzerland

**Keywords:** miRNA, isomiR, Deep sequencing, Endometrium, Implantation, Pig

## Abstract

**Background:**

Embryo implantation is a complex, synchronized process that requires establishment of a reciprocal dialogue between a receptive endometrium and developing blastocysts. Recently, microRNAs (miRNAs), known to modulate gene expression through post-transcriptional mechanisms, were implicated in regulation of early pregnancy events including maternal recognition of pregnancy and implantation. To characterize complex transcriptomic changes, expression of miRNAs in pregnant and cyclic endometria collected on days 12, 16 and 20 was analyzed using Illumina deep sequencing and analyzed with bioinformatic pipeline. Moreover, expression profiles of ten genes related to miRNA synthesis and transport such as *DROSHA*, *DGCR8*, *XPO5*, *DICER*, *TARBP2*, *TNRC6A*, and *AGO1*-*4* were determined.

**Results:**

Among genes involved in miRNA transport and synthesis *DROSHA*, *XPO5*, *DICER1*, *TARBP*, and *AGO1* expression was affected by the reproductive status. Moreover, DICER1 and AGO2 proteins were localized in luminal and glandular epithelium with immunofluorescence staining. Several hundred mature, canonical and non-canonical miRNAs were found to be expressed in the endometrial samples. Detailed analysis revealed that miRNA length variants, isomiRs, accounted for the vast majority of defined sequences. Both miRNA and isomiR of miR-140-3p were shown to affect expression of putative targets in endometrial stromal cells *in vitro*. Computational analysis of putative target genes for miRNAs differentially expressed (DE) between pregnant and cyclic animals resulted in lists of biological processes and regulatory pathways indicating their role in cellular development, cell cycle, immunological response and organismal development. Among predicted target genes for DE miRNAs, vascular endothelial growth factor (VEGF), progesterone and estradiol receptors (PGR, ESR1) and leukemia inhibitory factor (LIF) were found.

**Conclusions:**

This research revealed a repertoire of pregnancy-related miRNAs in porcine endometrium during initial stages of conceptus implantation and during the estrous cycle, and sheds light on mechanisms regulating miRNA-mediated gene expression at the maternal-conceptus interface.

**Electronic supplementary material:**

The online version of this article (doi:10.1186/s12864-015-2172-2) contains supplementary material, which is available to authorized users.

## Background

MicroRNAs (miRNAs) are small, non-coding regulatory RNAs that affect gene expression by partial complementary pairing with target mRNAs [1]. Transcribed from genomic DNA, pri-miRNAs containing a long hairpin are further processed in the nucleus to the pre-miRNA. Then, after maturation in the cytoplasm they can exert their function leading to translational repression and/or mRNA destabilization and degradation. These small RNAs are synthesized in a multi-step process that involves several specific proteins and enzymes [2]. First, pri-miRNAs are processed by a DROSHA/DGCR8 microprocessor complex subunit into 70–100 nt hairpin/stem-loop precursors (pre-miRNAs). Once exported from the nucleus by exportin-5 (XPO5), pre-miRNAs are cleaved by DICER1 to produce ~22 nt-long miRNA/miRNA* dimers. Final maturation of miRNAs and their proper functioning require assembly of a set of proteins/enzymes known as RISC (RNA-Induced Silencing Complex; [3]). DICER1 and its protein partners, HIV-1 transactivation response (TAR), RNA- binding protein 2 (TARBP 2), a trinucleotide repeat containing 6A (TNRC6A), and members of the Argonaute (AGO) family, are the main components of this complex. Although the generally-accepted mechanism assumes that only a guide strand is incorporated into the RISC and the opposite strand, known as miRNA* (miRNA-3p, passenger strand) is degraded, there is evidence that the latter can also be functional [4]. Finally, mature miRNAs guide Argonaute-containing complexes to target sites mainly in the 3’UTR region of the given transcript, which inhibits gene expression. Recent deep sequencing studies found that miRNAs exist as populations of variants (isomiRs) of different lengths and nucleotide composition [5, 6].

Studies in rodents [7–9] and in humans [10, 11] have demonstrated characteristic expression patterns of miRNAs in the pregnant uterus, implicating them not only in proper progression of pregnancy but also in implantation defects or spontaneous fetal loss. Chakrabarty et al. [7] showed that during the peri-implantation period in mice, expression of prostaglandin-endoperoxide synthase 2 (PTGS2), an enzyme crucial for prostaglandin (PG) synthesis, is regulated by miR-101, miR-144 and miR-199a*. Xia et al. [8] proved that activated blastocysts may influence endometrial expression of miR-320 during implantation in rats, while Wang et al. [11] provided evidence that miR-133a causes recurrent spontaneous abortion by reducing HLA-G expression in humans. Recently, Wessels et al. [12] using microarrays demonstrated differential patterns of canonical miRNA expression in the pregnant and non-pregnant uterus, as well as in normal or arrested trophoblasts on day 20 of pregnancy in pigs. Applying the same technique, Su and co-workers [13] analyzed miRNA expression in the porcine endometrium during embryo implantation (day15), placentation (day 26) and in mid-gestation (day 50), showing that some canonical miRNAs targeted the well-studied genes which are critical for placenta development in pigs.

Although a growing number of reports describe tissue- and time-specific miRNA expression in the pregnant and cyclic uterus in different species, there is still a lack of precise data about the endometrial profile of canonical and non-canonical miRNAs during the crucial periods of maternal recognition of pregnancy (days 11–12) and embryo implantation (days 13–24) in pigs. Therefore, identification of miRNAs as well as characterization of specific miRNA-mRNA interactions in the porcine endometrium is of great importance for understanding the role of these molecules in porcine reproduction. In the present study, by employing Illumina deep sequencing, we identified hundreds of miRNA sequences and their isomiRs, which by regulating their putative target genes may play a role during early pregnancy in the pig. Since generally rarer, 5’ end polymorphic isomiRs may represent a significant proportion of the population of some miRNAs we investigated *in vitro* whether 5’ isomiR of miR-140-3p with shifted seed is indeed capable of conferring differential targeting recognition and thus has different functions in the porcine luminal endometrium. Moreover, time- and reproductive status-specific changes in the expression profiles of ten synthesis/transport-related genes were evaluated in order to identify characteristic patterns of miRNA biosynthesis regulation.

## Results

### Expression of miRNA biosynthesis/transport related genes in the porcine endometrium

**Fig. 1:**
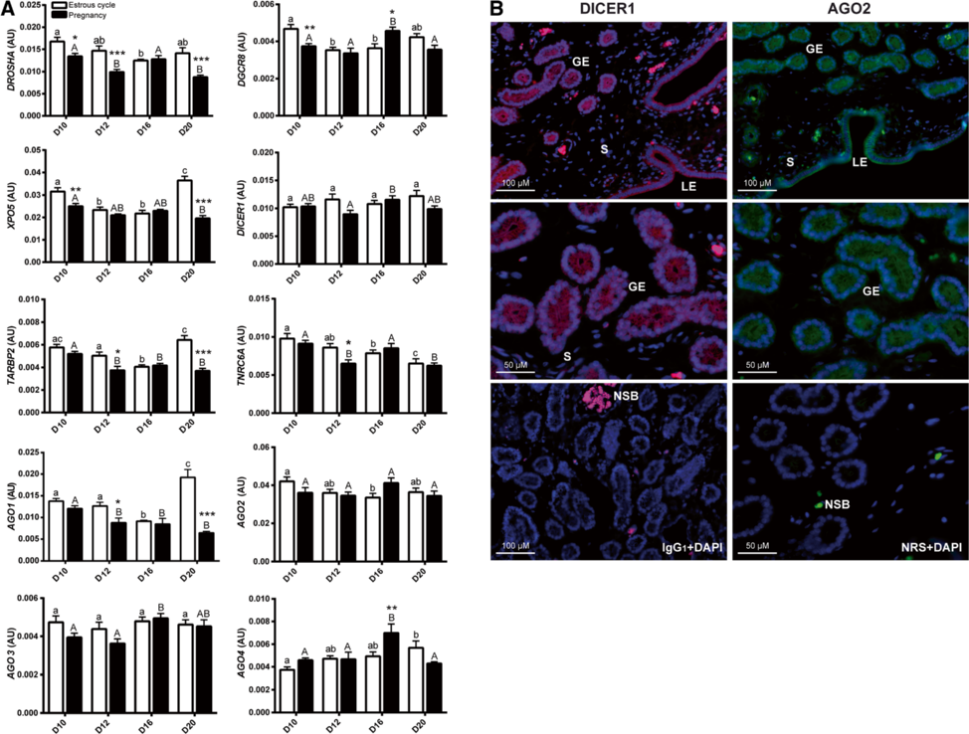
miRNA synthesis and transport related molecules are differentially expressed in the porcine endometrium. **a** Relative expression of ten genes related to miRNA synthesis/transport. Levels of *DROSHA, DGCR8, XPO5, DICER1, TARBP2, TNRC6A* and *AGO 1–4* genes are presented relatively to geometric means of *ACTB* and *PPIB*. Results are presented as boxplots with means ± SEM. Means with different superscripts differ significantly (small letters – the estrous cycle, capital letters – pregnancy). * *p* < 0.05, ** *p* < 0.01, and *** *p* < 0.001 indicate difference between reproductive states (estrous cycle *vs.* pregnancy) within the same day (n = 5-8 animals/group). AU – arbitrary units. **b** Microphotographs illustrating immunofluorescent staining of DICER1 (*red*) and AGO2 (*green*) proteins in the endometrial sections from D12 of pregnancy (*upper panel*) and D12 of estrous cycle (*middle panel*). Lower panel shows negative controls; staining with mouse IgG_1_ or NRS. DAPI was used for counterstaining of the nuclei (*blue*). NSB – non-specific binding (erythrocytes), GE – glandular epithelium, LE – luminal epithelium, S – stroma

Expression of analyzed genes (Fig. 1a) was assessed relatively to the geometric mean of the most stable genes – *ACTB* and *PPIB* (stability value = 0.142). The mRNA expression of *DROSHA* was affected by day (*p* = 0.0002) and reproductive status (*p* < 0.0001; day x status, *p* = 0.002). The highest *DROSHA* level in the cycle was observed on D10, and then decreased slightly reaching the lowest levels on D16 (*vs.* D10, *p* < 0.001)*.* During pregnancy, mRNA level for this gene was higher on D10 and on D16 (*vs.* D12, *p* < 0.05 and *vs.* D20, *p* < 0.001). In almost all within a day comparisons expression of *DROSHA* was greater in cyclic than pregnant endometria (*p* < 0.05) except day 16 where comparable levels were observed. *DGCR8* expression was affected by day (*p* = 0.005), but not the reproductive status (day x status, *p* = 0.0002). mRNA level of this gene was the highest on D16 of pregnancy in comparison to other days (*vs.* D10, *p* < 0.05; D12, *p* < 0.001 and D20, *p* < 0.01), as well when compared to the corresponding day of the estrous cycle (*p* < 0.01). In contrast, it was maintained lower on D10 of pregnancy (*vs.* D10 of the estrous cycle, *p* < 0.001). Expression of *XPO5* was significantly different between analyzed days (*p* < 0.0001) and dependent on reproductive status (*p* < 0.0001). In cyclic animals, the highest *XPO5* mRNA level was observed on D20 (*vs.* D10, *p* < 0.05; D12 and D16, *p* < 0.001). High level of this gene was also noted on D10 when compared to D12 and D16 (*p* < 0.001). On D10 and D20, higher *XPO5* mRNA expression was noted in cyclic *vs.* pregnant animals (*p* < 0.01 and *p* < 0.001, respectively). During pregnancy *XPO5* expression was maintained at comparable levels except significant decrease on D20 (*vs.* D10, *p* < 0.05). *DICER1* expression was affected by reproductive status (*p* = 0.049) but not day (day x status, *p* = 0.047). During the estrous cycle mRNA level of this gene was maintained at similar level. However, in pregnant animals mRNA expression of *DICER1* was increased on D16 *vs.* D12 (*p* < 0.05). *TARBP2* level was affected by day (*p* < 0.0001) and reproductive status (*p* < 0.000; day x status, *p* < 0.0001). In endometria from cyclic animals expression of this gene was higher on D20 *vs.* D12 (*p* < 0.01) and *vs.* D16 (*p* < 0.001). During pregnancy, *TARBP2* expression was maintained high on D10 when compared to other days of pregnancy (*vs.* D12 and D20, *p* < 0.001; *vs.* D16, *p* < 0.05). Significantly lower expression of this gene was observed on D12 and D20 in pregnant animals *vs.* cyclic counterparts (*p* < 0.05 and *p* < 0.001, respectively). *TNRC6A* expression was affected by day (*p* < 0.0001) but not the reproductive status. In cyclic animals *TNRC6A* expression was greatest on D10 (*vs.* D16, *p* < 0.05) and subsequently decreased to reach the lowest levels on D20 (*vs.* D10, *p* < 0.001 and D12, *p* < 0.05). In pregnant animals, the lowest expression of *TNRC6A* was observed on D12 *vs.* D10 and D16 (*p* < 0.01 and *p* < 0.05, respectively), and when compared to D12 of the estrous cycle (*p* < 0.05). *AGO1* gene expression was affected by day (*p* < 0.0001) and the reproductive status (*p* < 0.0001; day x status, *p* < 0.0001). Lower *AGO1* mRNA expression observed on D16 of estrous cycle when compared to D10 (*p* < 0.01), D12 (*p* < 0.05) and D20 (*p* < 0.001). On the other hand, the highest mRNA expression of *AGO1* during estrous cycle was indicated on D20 when compared to other days (*p* < 0.001). During pregnancy, *AGO1* mRNA level was highest on D10 and gradually decreased on subsequent days (*vs.* D12 and D16, *p* < 0.05), reaching the lowest levels on D20 (*p* < 0.001). Expression of this gene was decreased on D12 and D20 of pregnancy when compared to corresponding days of the estrous cycle (*p* < 0.05 and *p* < 0.001, respectively). Expression of *AGO2* was neither affected by day nor by the reproductive status (day x status, *p* = 0.035). Highest level of this gene in cyclic animals was observed on D10 *vs.* D16 (*p* < 0.05). During pregnancy mRNA level of *AGO2* did not differ significantly. Expression of *AGO3* gene was maintained at comparable levels during the estrous cycle, while during pregnancy increased expression was observed on D16 when compared to other days (*vs.* D10, *p* < 0.05 and D12, *p* < 0.01; day x status, *p* = 0.03). Changed expression of *AGO4* on tested days (*p* = 0.0022) of the estrous cycle and pregnancy was indicated. Low expression of this gene in endometria from cyclic gilts was observed on D10, which gradually increased reaching the statistical significance on D20 (*vs.* D10, *p* < 0.05). During pregnancy, *AGO4* level was higher on D16 in comparison to other days (*vs.* D10 and D20, *p* < 0.001; D12, *p* < 0.01), and corresponding day of the estrous cycle (*p* < 0.01).

### Immunolocalization of DICER1 and AGO2 in the porcine endometria

Immunostaining for DICER1 and AGO2 was detectable in the uterine sections from all days of the estrous cycle and early pregnancy examined. Specific binding of antibodies for both proteins, showed in Fig. 1b , was limited to luminal (upper panel) and glandular epithelium (middle panel). Endometrial stroma was free from staining, likewise negative controls (bottom panel).

### Overview of the sequences generated by Illumina sequencing

Average raw read counts obtained from Illumina sequencing (Additional file 1: Table S1) ranged from 6,603,153 on D16 of pregnancy up to 9,200,516 on D20 of pregnancy. For instance, for D20 of the estrous cycle, number of reads, after applying phred quality score filtering [14] (phred score >20 for each base) was reduced from 6,605,189 to 3,210,430. For 527,852 sequences it was not possible to find adapter sequences, which resulted in reduction of total read count to 2,682,578. Afterwards, sequences with length below 17 nt and sequences containing unknown nucleotides (ambiguous results of A, C, G or T annotation) were discarded. Remaining 2,645,026 valid sequences were collapsed, which resulted in the final number of 51,868 unique sequences. In general, sequences obtained after pre-analysis for all libraries where characterized by length ranging from 17–31 nt with a predominant length of 22 nt, phred quality score from 23 to 40 and GC bases content per sequence around 40 % (Additional file 2: Figure S1).

### Characterization of isomiRs detected in the porcine endometrium

**Fig. 2:**
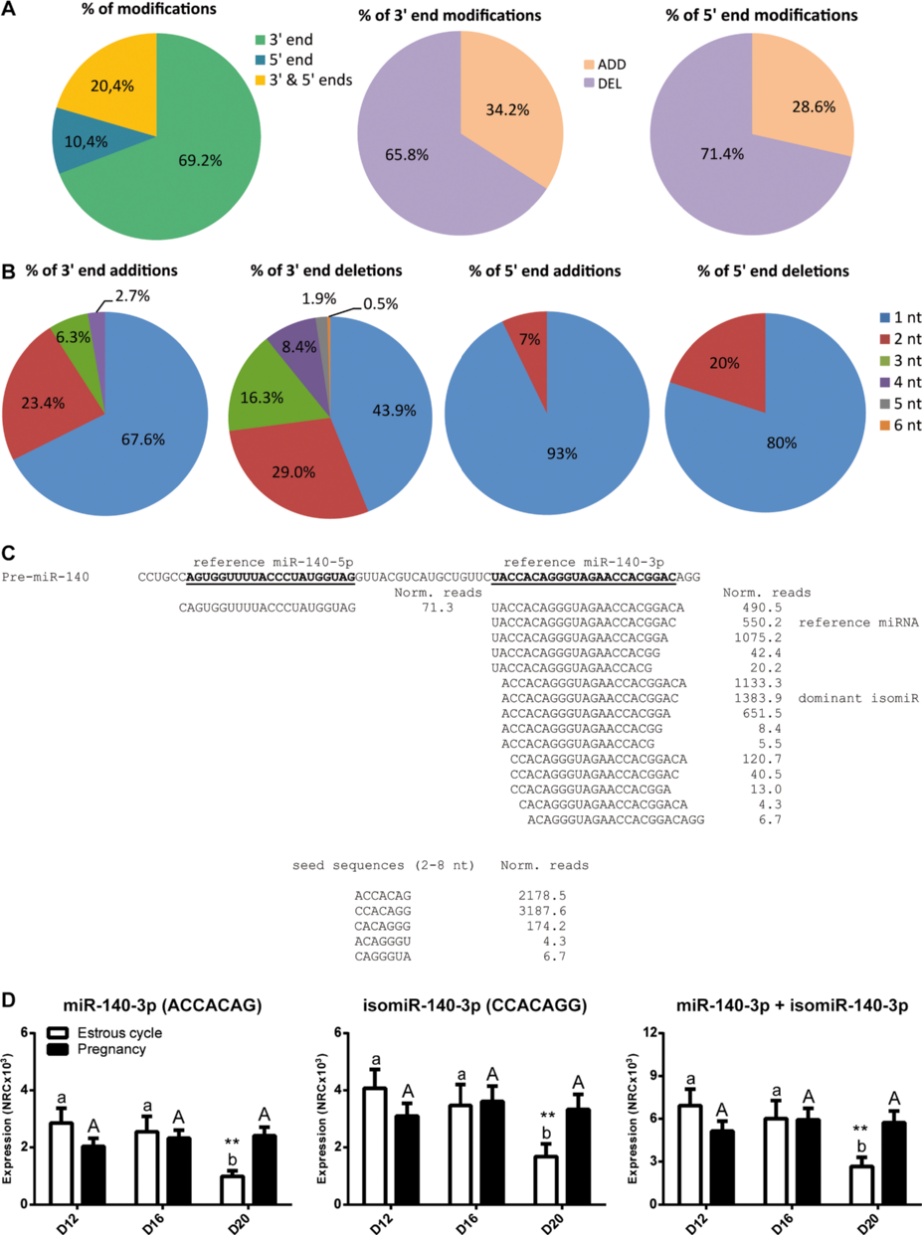
Modifications of miRNA length expressed in the porcine endometria collected on different days of the estrous cycle or pregnancy. **a** Percent of modifications affecting either 3’ end, 5’ end or both ends; **b** Percent of additions and deletions on 3’ end, and percent of additions and deletions on 5’ end. **c** Mean values of normalized read counts from all groups for reference/canonical miR-140-3p or its isomiRs and sum of normalized read counts for each seed sequence. **d** Expression of reference/canonical miR-140-3p (seed sequence ACCACAG) and its 5’ isomiR with seed sequence CCACAGG. miRNA/isomiR expression on different days of the estrous cycle and pregnancy (*n* = 4-6 animals/group) is presented as mean of normalized reads counts (NRC) ± SEM. Two-way ANOVA analysis with Bonferoni post-hoc test was performed on log-transformed values to find statistical differences. Means with different superscripts differ significantly (small letters – the estrous cycle, capital letters – pregnancy). ** *p* < 0.01, indicate difference between reproductive states (estrous cycle *vs.* pregnancy) within the same day

By aligning the valid, filtered sequences against the porcine genome (Ensembl Sscrofa10.2) [15], miRBase v. 18 [16], and nucleotide collection in NCBI BLASTn [17], 873 sequences (100 % homology) were annotated as mature miRNAs and isomiRs, found in pig or in other mammalian species (Additional file 3: Table S2). Analysis aimed to characterize isomiRs were performed only for the sequences known in the pig, which could be divided into the following groups: i) canonical, mature miRNAs with isomiRs (102 and 433 sequences, respectively), ii) only mature, canonical miRNAs (26 sequences); iii) only isomiRs, without canonical form present in the dataset (96 sequences). In regard to the last group, additional step was performed and isomiRs were described based on the presence of mature, canonical miRNAs in the miRBase v. 20 (37 out of 96 sequences were analyzed). Some sequences were excluded from further isomiR characterization since it was not possible to annotate them to one canonical miRNA (Additional file 4: Figure S2). Finally, 470 isomiRs known in pig were included in the subsequent analysis. miR-10a-5p (20 isomiRs), miR-21 (17) and miR-140-3p (14) showed the highest numbers of isomiRs. Interestingly, numbers of reads for some isomiRs were more abundant than for mature, canonical miRNAs (Additional file 3: Table S2, e.g. ssc-miR-30a-5p, miR-30d, and miR-99a). Modifications of the length were related to additions (ADD) and deletions (DEL) of nucleotides, present at 3’ or 5’ end, or at both ends simultaneously (Fig. 2a). Modifications restricted to 3’ end accounted for 69 % of modifications (325 sequences from 470), while modifications on 5’ end and both ends accounted for 49 and 96 sequences, respectively (Fig. 2a). The most frequent modification on 3’ and 5’ ends were DEL (66 and 71 %, respectively; Fig. 2a) with dominant DEL of 1 nt (44 and 80 % of deletions, respectively; Fig. 2b). The least frequently occurring modifications on the 3’ end were: ADD of 4 nt (2.7 %) and DEL of 6 nt (0.5 %), while on the 5’ end modifications applied only to two nucleotides (Fig. 2b). miRNAs with the greatest number of modifications such as miR-10a-5p, miR-21 and miR-140-3p accounted for about 40–50 % of mean read counts for each group. Moreover, isomiR for miR-21, with ADD of cytosine on 3’ end, had the highest number of reads (averaged for all libraries) among all detected isomiRs.

### DE miRNAs and isomiRs in endometrium of pregnant and cyclic pigs

Normalization and statistical analysis performed with EdgeR [18] indicated that from 873 sequences, 223 were differentially expressed between all days of the estrous cycle (C) and pregnancy (P; Additional file 5: Table S3). Log2-transformed fold change values ranged from −4.87 up to 8.5. The highest number of DE miRNAs was found between PD20 and CD20. Surprisingly no miRNA reached the significant FDR level for comparison between the estrous cycle and pregnancy on D12.

Furthermore, for some sequences only isomiRs or mature forms were differentially expressed, but for others both miRNAs and isomiRs showed altered patterns of expression. For instance, miR-182 was represented in our dataset by canonical miRNA and 9 template 3’ isomiRs of which only 5 showed significantly changed expression in pregnant *vs.* cyclic animals (Additional file 5: Table S3). Among miRNAs showing the highest number of isomiRs, miR-140-3p was represented by canonical form, 4 isomiRs of 3’ end and 10 isomiRs with modified 5’ or both 5’ and 3’ ends (Fig. 2c). Interestingly, canonical miR-140-3p and two 3’ isomiRs showed similar expression pattern at different days of the estrous cycle or pregnancy (PD20 *vs.* CD20, CD20 *vs.* CD12 and CD16; Additional file 5: Table S3). Additionally, several miRNAs/isomiRs belonging to the same family, such as miR-23a/b or miR-148a/b also fallowed similar expression patterns (Additional file 5: Table S3; Additional file 6: Figure S3). To check if miR-140-3p and its isomiRs bearing the same sequence are differentially expressed and preserve expression profile of canonical sequence a sum of normalized read counts was subjected to further statistical analysis. Interestingly, level of 3’isomiRs and canonical miR-140-3p (seed sequence ACCACAG) was higher on PD20 *vs.* CD20 (*p* < 0.01), CD20 *vs.* CD12 and *vs.* CD16 (*p* < 0.001 and *p* < 0.01, respectively; Fig. 2d). Moreover, highly expressed 5’ isomiRs (seed sequence CCACAGG) fallowed similar expression pattern that was also true for combined read counts for both seed sequences (Fig. 2d).

**Fig. 3:**
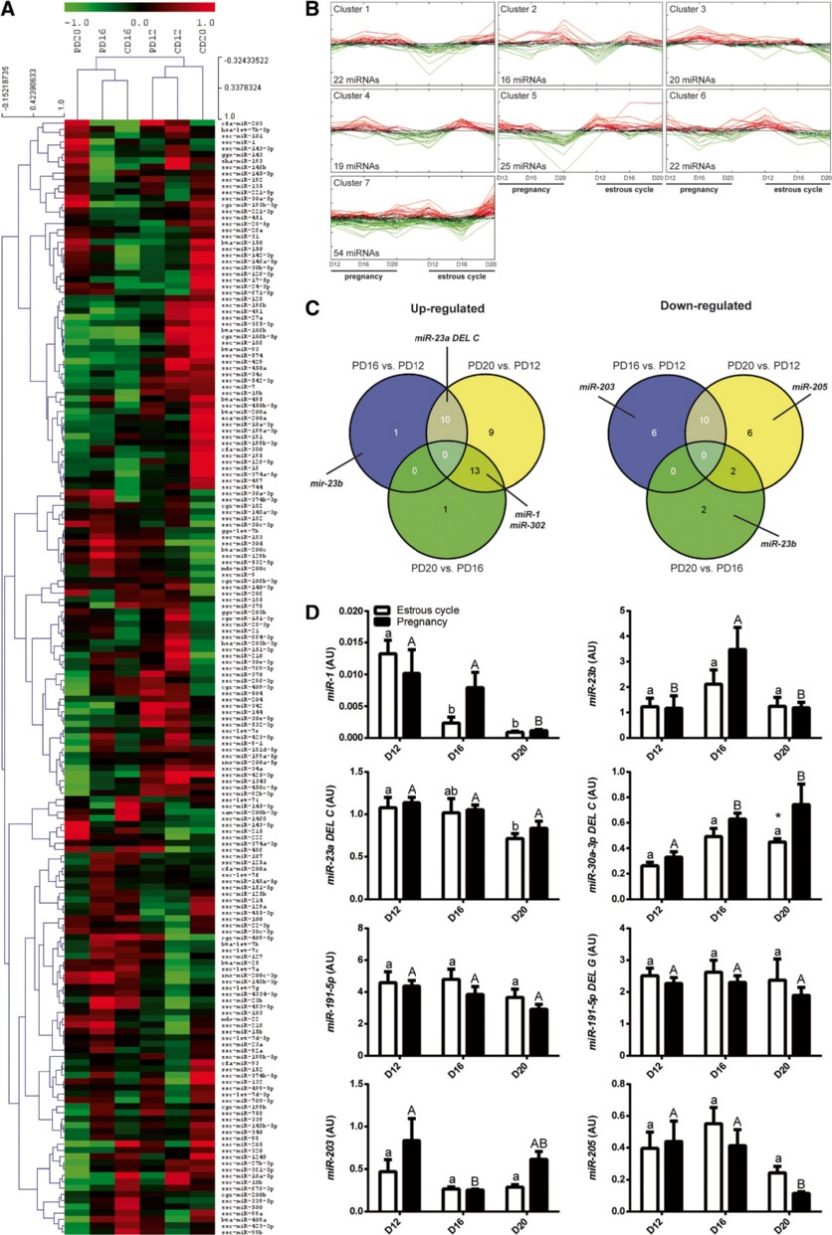
miRNAs/isomiRs showing differential expression on D12, D16 and D20 of the estrous cycle and early pregnancy. **a** Dendrogram representing results of unsupervised hierarchical clustering (HCL) created with Pearson correlation coefficient. Rows indicate single miRNAs, while columns represent different days of the estrous cycle and pregnancy. **b** Clusters generated by Self Organizing Tree Algorithm (SOTA) representing expression profiles of mature, canonical miRNAs expressed in the porcine endometrium of pregnant and cyclic pigs. **c** Venn diagrams demonstrating relations between DE miRNAs and isomiRs for selected comparisons during pregnancy. Marked up- and down-regulated miRNAs were chosen for microarray validation by real-time RT-PCR. **d** Relative expression of DE miRNAs selected for validation of Illumina sequencing, using stem-loop real-time RT-PCR. miRNA expression on different days of the estrous cycle and pregnancy (*n* = 7-9 animals/group) is presented relative to the reference let-7d-3p. Two-way ANOVA analysis with Bonferoni *post-hoc* test was performed to find statistical differences. Results are presented as boxplots with means ± SEM. Means with different superscripts differ significantly (small letters – the estrous cycle, capital letters – pregnancy). * *p* < 0.05, indicate difference between reproductive states (estrous cycle *vs.* pregnancy) within the same day. AU – arbitrary units

Unsupervised hierarchical clustering (HCL) and SOTA clustering were performed using only mature/canonical porcine or mammalian miRNAs (178 miRNAs out of 873 sequences). More similar miRNA expression pattern was observed for PD12 and CD12, as well between PD16 and CD16. The biggest distance calculated with Pearson correlation coefficient was noted between PD20 and CD20 (Fig. 3a). According to the SOTA, DE miRNAs were grouped into 7 different clusters (Fig. 3b; Additional file 7: Table S4). Several characteristic profiles could be observed. For example, in cluster 2 miRNAs grouped (*e.g.* miR-1), which levels were increased on PD20 and CD16, and lower on PD16 and CD12 when compared to other analyzed days. Cluster 4 consisted of 19 miRNAs (*e.g.* miR-23b), which expression was elevated on PD16 and CD16, and lower on CD12. Cluster 5 contained such miRNAs as miR-205 and miR-34a, showing lower levels on PD20 in comparison to CD20. For cluster 7 (54 miRNAs), expression profiles were characterized by elevated miRNA levels on CD20 when compared with all other periods analyzed. Venn diagrams (Fig. 3c) showed thirteen common miRNAs up-regulated on PD20 in comparison to PD12 and PD16 (*e.g.* miR-1, miR-302). Moreover, ten miRNAs were found to be up-regulated (*e.g.* miR-23a DEL C) as well as down-regulated (*e.g.* miR-342) on PD20 and PD16 comparing to PD12. On the other hand, there was no miRNAs commonly expressed between all comparisons presented, and only one miRNA was found to be specifically up-regulated for comparison PD16 *vs.* PD12 (miR-23b) and PD20 *vs.* PD16 (miR-143).

To confirm miRNA profiles of several DE miRNAs found in sequencing, stem-loop real-time RT-PCR was performed. miRNA expression profiles are presented relatively to the reference miRNA let-7d (stability value = 0.303) in Fig. 3d. Similar patterns as observed in sequencing data were observed for most of miRNAs. Higher levels of miR-23b were observed on PD16 *vs.* PD12 and PD20 (*p* < 0.01). miR-203 expression was elevated on PD12 when compared to PD16 (*p* < 0.05). Increased levels of miR-30a-3p isomiR (3’deletion of C) were observed on PD20 *vs.* PD12 (*p* < 0.01) and on PD20 *vs.* CD20 (*p* < 0.05). Expression pattern for miR-205 was only confirmed for analyzed days of pregnancy, with decreased levels on PD20 *vs.* PD12 and PD16 (*p* < 0.01). PCR Miner analysis failed for miR-302, since expression of this miRNA was at the detection limit of the real-time RT-PCR method. Nonetheless, when monitored in detail, expression of miR-302 was detectable exclusively in samples from PD20, as it was observed in NGS data, except two samples with very few reads from CD20. Decreased levels of canonical miR-191-5p and its isomiR - miR-191-5p DEL G (3’ deletion of G) found to be significant on PD20 *vs.* CD20 in sequencing were not confirmed by real-time RT-PCR. Using specific assays we also detected miR-1 and isomiR miR-23a-3p DEL C (3’ deletion of C), however their expression did not follow sequencing profile.

### Functional annotation and target prediction for DE miRNAs

**Fig. 4:**
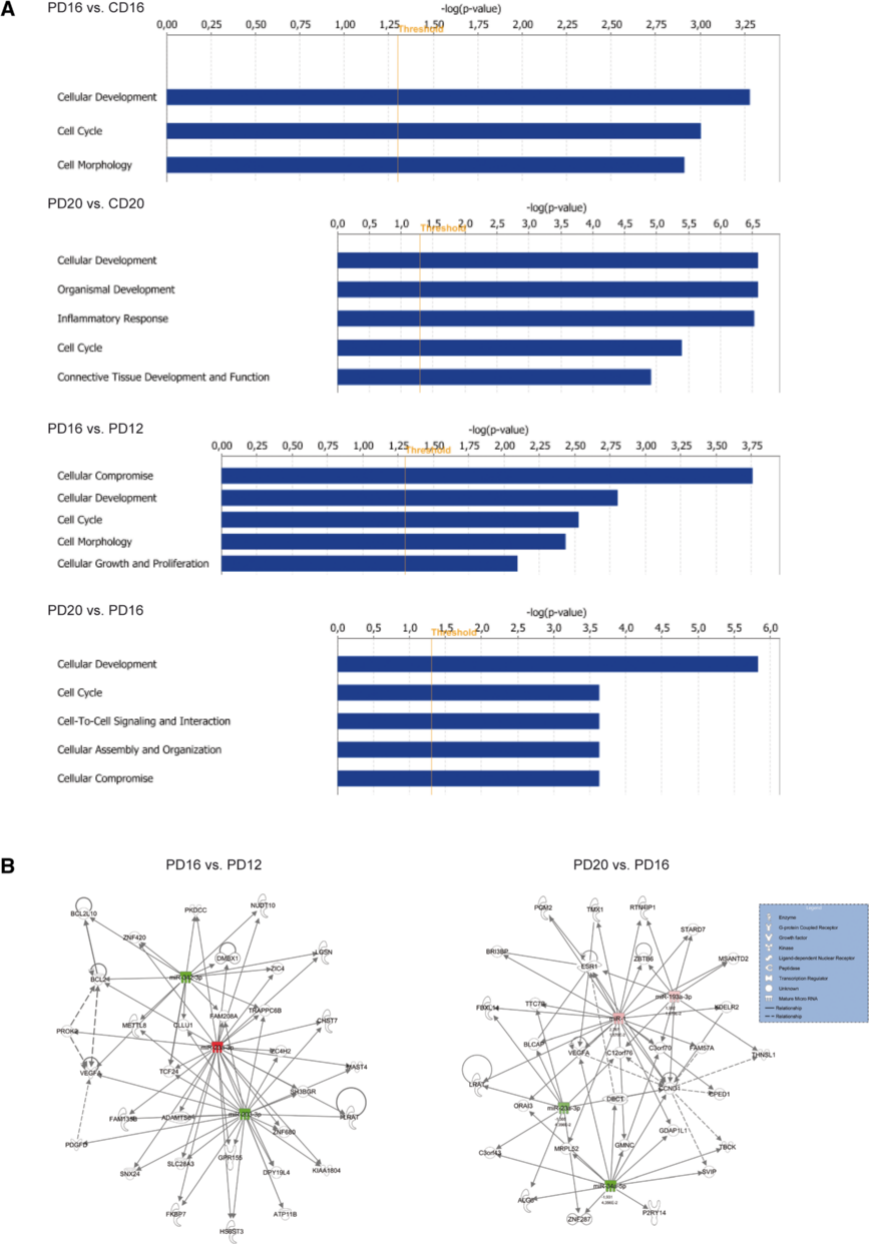
miRNAs showing differential expression in porcine endometrium classified into various functions and pathways by Ingenuity Pathway Analysis (IPA). **a** Top molecular functions enriched with DE miRNAs for PD20 *vs*. CD20, PD20 *vs*. PD16, PD16 *vs*. CD16, PD16 *vs.* PD12 and CD16 *vs*. C12 comparisons. The X-axis represents score for the likelihood [−log (Benjamin-Hochberg *p*-value < 0.05)] that genes belonging to a specific function category are affected in the specific comparisons. **b** Networks representing molecules of interest differentially regulated in selected comparisons. The network is displayed graphically as nodes (miRNAs/genes/genes products) and edges (biological relationship between nodes). The node color intensity indicates the fold-change expression of miRNAs; with red representing up-regulation and green down-regulation of miRNAs. The log2 fold change value and FDR for individual miRNAs is indicated under each node. The shapes of nodes indicate the functional class of the gene product and the lines indicate the type of interaction, explained in the legend. All connections were integrated into the computationally generated networks on the basis of the evidence stored in the IPA knowledge memory indicating relevance for these networks

Unfortunately, most (if not all) of the freely available tools for miRNA-mRNA interaction predictions allow input of miRNA names, but not sequences; thus cannot be used to adequate analysis of isomiRs-mRNA interactions. Therefore, for biological processes and target prediction analysis 61 DE miRNAs with human identifiers were used in Ingenuity Pathway Analysis (IPA). To expose more significant changes and processes in which DE miRNAs could be involved during the estrous cycle and pregnancy we selected PD16 *vs.* CD16, PD20 *vs.* CD20, PD16 *vs.* PD12 and PD20 *vs.* PD16 comparisons (Fig. 4a). Top enriched processes for PD16 *vs*. CD16 comparison were those associated with cellular development (*p* = 5.25E-04), cell cycle (*p* = 9.91E-04), cell morphology (*p* = 1.22E-03) and mainly miR-205-5p was involved in this actions (Additional file 8: Table S5). When compared PD20 *vs*. CD20 the top processes affected were those related to cellular development (2.58E-07 – 4.89E-02), organismal development (2.58E-07 – 4.89E-02) and inflammatory response (2.97E-07 – 4.89E-02). Several miRNAs, which were related to abovementioned actions included miR-34a-5p, miR-205-5p, miR-30c-5p, miR-17-5p. Cellular compromise (1.75E-04), cellular development (1.57E-03 – 3.03E-02), cell cycle (2.97E-03), and cell morphology (3.67E-03) were among mostly enriched processes for PD16 *vs*. PD12 comparison. For PD20 *vs*. PD16 comparison, miR-23a-3p, miR-34a-5p, miR-1 were indicated as central molecules affecting cellular development (1.47E-06 – 4.85E-02), cell cycle (2.33E-04 – 1.63E-03), cell-to-cell signaling and interaction (4.67E-04 – 2.33E-04) or embryonic development (1.17E-03 – 4.29E-02). Molecular pathways for selected comparisons were automatically generated in IPA and two examples are presented in Fig. 4b.

Several miRNA-mRNA interactions revealed by the software indicated possible effects of miRNAs action on genes crucial in pregnancy related events (Table 1). For example, miR-23b was found to regulate progesterone (PGR) and estrogen (ESR1) receptors, interleukin 6 receptor (IL6R) and growth factors, such as fibroblast growth factor 2 (FGF2). Interestingly, vascular endothelial growth factor A (VEGFA) was found to be target for several miRNAs, including miR-1, miR-203, miR-23b and miR-205. Among genes found to be putative targets for miR-27a were LIF and its receptor (LIFR), IL6R or insulin growth factor 1 (IGF1).

**Table 1:** Selected pregnancy associated target genes for differentially expressed miRNAs in the porcine endometrium

Functional category	Target gene^a^	DE miRNAs
Prostaglandin signaling and metabolism	PTGS2	miR-758, miR-16, miR-320, let-7a, miR-199a-3p, miR-203
	PTGER2	miR-429
	PTGFR	miR-23b, miR-92b-3p, miR-205, miR-429
	PTGIS	miR-449a, miR-150, miR-200a
	PTGIR	miR-1, miR-205
Inflammatory mediators	IL6	let-7e, miR-365-3p, miR-574
	IL6R	miR-200a, miR-27a/b, miR-24-3p, miR-342, miR-23b, miR-495, let-7a, miR-17, miR-449a
	LIF	miR-106b-5p, miR-27a/b, miR-17-5p, miR-574
	LIFR	miR-1, miR-142-3p, miR-342, miR-365-3p, miR-203, miR-30d, miR-27a, miR-23b, miR-205, miR-191, miR-199a-3p
Growth factors, receptors and related proteins	IGF1	miR-1, miR-495, miR-186, miR-486, miR-23b, miR-27a, miR-380, miR-30d, miR-361-5p, miR-16, let-7a
	IGF1R	let-7a, miR-100, miR-200a, miR-186, miR-16, miR-30d, miR-96, miR-495, miR-375
	IGFBP1	miR-140-3p, miR-205, let-7a
	IGFBP3	miR-374a-5p, miR-449a, miR-1249, let-7a
	IGFBP5	miR-1, miR-24-3p, miR-146a-5p, miR-193b-3p, miR-203, miR-27a
	VEGFA	miR-342, miR-200a, miR-1, miR-1249, miR-186, miR-449a, miR-16, miR-205, miR-203, miR-150, miR-374a-5p, miR-361-5p, miR-17-5p, miR-106b, miR-23b, miR-429
	KDR	miR-16, miR-200b, miR-23b
	Flt1	miR-96, miR-142-3p, miR-200b, miR-200a, miR-374a-5p, miR-193b-3p, miR-17-5p, miR-106b
	FGF7	miR-27a, miR-186, miR-495, miR-199b-3p, miR-361-5p, miR-140, miR-16, miR-132, miR-486, miR-17, miR-429
	FGF2	miR-16, miR-23b, miR-203, miR-17
	SPRED1	miR-1, miR-96, miR-16, mniR-199a-3p, miR-132, miR-142-3p, miR-200c-3p, miR-17-5p, miR-106b, miR-449a, miR-126
Other	Wnt4	miR-16, miR-186, miR-24-3p, miR-429
	Wnt5a	miR-129b, miR-140-3p, miR-374b-5p, miR-200a, miR-92b-3p, miR-365-3p, miR-186, miR-30d, miR-23b, miR-203, miR-205, miR-574
	Wnt7a	miR-361-5p, miR-16
	CTNNB1	miR-150, miR-200a
	CDH1	miR-495, let-7a, miR-23b
	HoxA10	miR-27a, miR-374b-5p, miR-16
	ESR1	miR-1, miR-17-5p, miR-20a, miR-203, miR-129b, miR-193, miR-106b, miR-23b
	PGR	miR-342, miR-129b, miR-96, miR-23b, miR-140-3p, miR-495

^a^Target prediction for DE miRNAs was done using IPA and miRWalk, which incorporate information from several algorithms such as: miRanda, TargetScan, miRDB, RNA22, PICTAR or DIANA-microT and miRwalk. miRNA target genes identified by at least three of these algorithms were considered for analysis

**Fig. 5:**
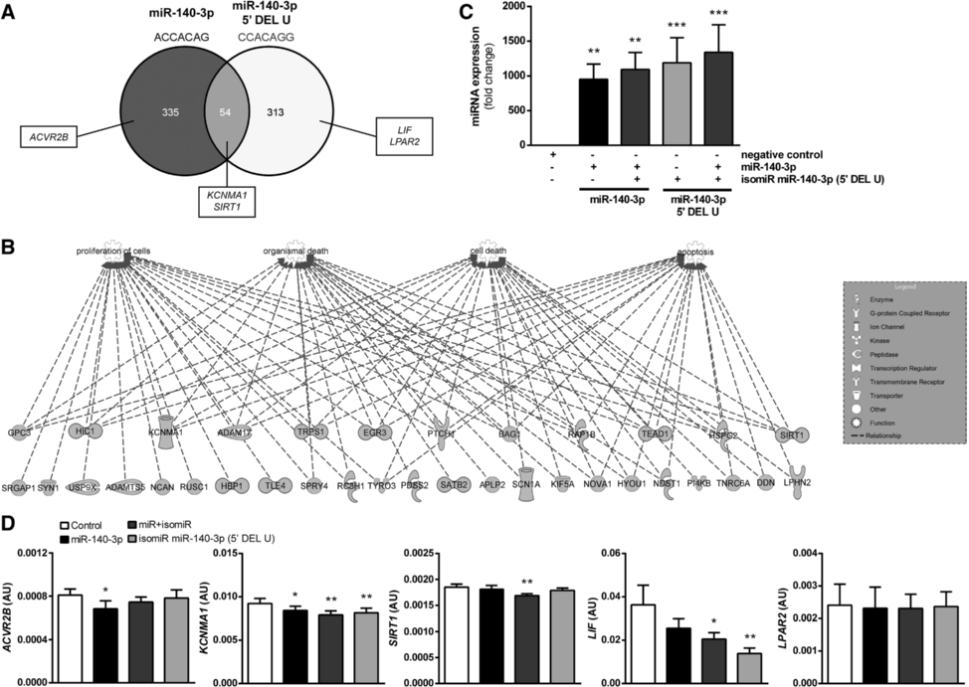
isomiRs work cooperatively with canonical miRNAs to affect putative targets gene expression in endometrial stromal cells. **a** Venn diagrams demonstrating relations between targets identified for canonical and non-canonical miR-140-3p harboring different seed sequences. **b** Top functional annotations organized into network representing common target mRNAs regulated by canonical and non-canonical miR-140-3p. The network is displayed graphically as nodes (genes/genes products) and edges (biological relationship between nodes). The shapes of nodes indicate the functional class of the gene product, explained in the legend. All connections were integrated into the computationally generated networks on the basis of the evidence stored in the IPA knowledge memory indicating relevance for these networks. **c** Fold change expression of miR-140-3p and its 5’ isomiR mimic transfected into endometrial stromal cells collected on D16 of pregnancy (*n* = 6) is compared to negative control. let-7d-3p was used as reference for miR-140-3p and isomiR miR-140-3p 5’ DEL U expression. Mimics were used at 50 nM dose when added separately or 25 nM dose each when added simultaneously. **d** Relative expression of canonical and non-canonical miR-140-3p putative target genes after mimics transfection into endometrial stromal cells from D16 of pregnancy (*n* = 4-6). Levels of *ACVR2B, KCMA1, SIRT1, LIF* and *LPAR2* genes are presented relatively to geometric means of *HPRT1* and *ACTB*. Results are shown as boxplots with means ± SEM. Asterisks indicate significant differences, in comparison to the negative control (50 nM; miRNA negative control mimic). *p < 0.05; **p < 0.01; ***p < 0.001

Overlapping expression of miRNA and isomiRs with different seed sequence found for miR-140-3p prompt us to predict putative targets for both seed sequences using TargetScanHuman version 6.2 or TargetScanHuman custom version 5.2 [19]. Fifty four common targets for canonical and non-canonical miR-140-3p (Fig. 5a) were found to regulate processes related to cell proliferation (23 molecules, *p* = 2.52E-03), apoptosis (19 molecules, *p* = 1.38E-03), cell differentiation (18 molecules, *p* = 3.72E-04), vasculogenesis (8 molecules, *p* = 5.95E-03), angiogenesis (7 molecules, *p* = 1.64-E02) or estrous cycle (2, *p* = 1.59-E02) as indicated by IPA (Fig. 5b, Additional file 9: Table S6).

### Regulation of gene expression by miR-140-3p and its 5’ isomiR

Since miR-140-3p was found to be differentially expressed in our data set and read count for its 5' isomiR was about three times higher than canonical one we decided to test them further to answer the question whether sequence modifications of 5’ end of the canonical miRNA may have a significant impact on putative target gene expression in porcine endometrial cells during pregnancy. We analyzed the effect of forced overexpression of porcine miR-140-3p and its isomiR (5’ DEL U) on mRNA levels of several putative targets *in vitro*. miRWalk [20] and custom version of TargetScanHuman custom version 5.2 [19] were used to find putative targets for studied molecules, respectively. By generating Venn Diagram, we selected several genes, which were common or specific predicted targets for miRNA or isomiR (Fig. 5a).

Because TargetScanHuman and TargetScanHuman Custom consider matches to annotated human UTRs and their orthologs in other species but not in *Sus scrofa*, we also compared 3’UTRs of five selected genes using the Sscrofa 10.2 genome assembly and the BLASTn search function. At least one highly conserved site was identified for *ACVR2B*, *KCNMA1*, *SIRT1*, *LIF*, and *LPAR2* (Additional file 10: Figure S4).

Transfection of miR-140-3p and its isomiR proved to be efficient in primary porcine stromal cells (Fig. 5c). As demonstrated on Fig. 5d, expression of *ACVR2B* (target for miRNA) was decreased in cells exposed to miR-140-3p (*p* < 0.05). *KCNMA1*, predicted as a common putative target for miRNA and isomiR, was downregulated by all treatments (miR, *p* < 0.05; isomiR and miR + isomiR, *p* < 0.01). Another common target *SIRT1* was affected only when miR and isomiR were added simultaneously to the culture (*p* < 0.01). Expression of *LIF*, predicted target for 5 ’isomiR was decreased in cells transfected either with isomiR alone (*p* < 0.01) or miR-140-3p and isomiR added simultaneously (*p* < 0.05). Interestingly, expression of *LPAR2* was not affected by any treatment.

## Discussion

Endometrial receptivity acquisition, associated with altered gene expression, is essential for attachment of the conceptus and its firm adhesion to the luminal epithelium [21]. Recently, miRNAs have been recognized as substantial participants in pregnancy-associated events [7–11]. Here, we showed expression profile of genes involved in miRNA synthesis and transport and we report a repertoire of miRNAs and isomiRs identified in porcine pregnant and cyclic endometria by deep sequencing analysis. A bioinformatic pipeline was developed to analyze raw data and annotate sequences to miRNAs as well as to define *in silico* a potential role of miRNAs expressed in the porcine endometrium. *In vitro* experiments proved that isomiRs, *i.e.* miR-140-3p 5’ DEL U, are able to work cooperatively with canonical miRNAs to affect putative targets gene expression in porcine endometrial stromal cells.

Recently, we demonstrated pregnancy stage-dependent expression profiles of ten genes involved in miRNA synthesis and transport in porcine conceptuses and trophoblast [22]. Here, we expanded our investigation into expression profiles of *DROSHA*, *DGCR8*, *XPO5*, *DICER1*, *TARBP2*, *TNRC6A*, *AGO1*-*4* in the porcine endometrium across the estrous cycle and early pregnancy. It has been shown that estrogens and progesterone may regulate expression of miRNA-synthesis-related genes as well as miRNA expression [23-25]. Bhat-Nakshatri et al. [23] proposed that this regulation may occur through steroid receptor-promoter interactions, with direct binding of ESR1 to the regulatory regions of miRNAs or mRNA encoding genes containing miRNAs. Simultaneous estrogen and progesterone treatment in mice increased expression of *DROSHA, DGCR8, XPO-5* and *DICER1* [25]. It was shown that this modulation occurs through cognate steroid receptors, since treatment with the respective antagonists, ICI 182,780 and RU-486, reversed the stimulatory effect of these hormones on the expression of synthesis/transport-related genes. Characteristic, status dependent profiles of genes involved in miRNA synthesis and transport observed in the present study suggest possible steroid hormone regulations.

Pre-ovulatory follicles in pigs intensely synthesize estrogen [26], which triggers the luteinizing hormone (LH) surge and prepares the female reproductive tract for conception. Elevated *XPO5, TARBP2*, *AGO1* and *AGO4* levels around the time of ovulation coincide with increased estradiol levels in blood, whereas progesterone is maintained at a low level. On the other hand, by D16 gradually decreasing expression of *DROSHA*, *DGCR8*, *XPO5*, *TARBP2, TNRC6A* and *AGO1* may be linked to reduced production of progesterone by CL in response to the luteolytic signal of endometrial PGF2α [27]. During pregnancy, porcine embryos produce and release in a biphasic manner large amounts of estrogen around D11-D12 and again between D15 and D30 [28, 29]. Although it is likely that endometrial gene expression during pregnancy is influenced by embryonic estrogen, concepti also produce cytokines such as IFN-γ and IFN-δ [30, 31], with the highest levels found on D16 of pregnancy [30]. After progesterone priming, IFNs alone or together with estrogen may affect expression of many genes in the peri-implantation uterus [32, 33], including those involved in miRNA synthesis and transport. In the present study, levels of *DROSHA, DGCR8, TNRC6A* and *AGO4* in pregnant animals were lower on D12 and D20 compared to D16. Moreover, expression of *DROSHA, TARBP2* and *AGO1* was also lower on D12 and D20 of pregnancy in comparison to corresponding days of the estrous cycle. Although the miRNA synthesis pathway in animals and plants has been well researched over the past decade, many questions have yet to be answered, including those about involvement of particular miRNA synthesis and transport related proteins in generation of non-canonical miRNAs.

Recent studies applying deep sequencing in the pig were mainly focused on profiling miRNA expression in regard to muscle development or fat deposition [34, 35]. Since early pregnancy events not only affect embryonic development but also the fate and quality of progeny after birth [36], it is of great importance to trace the molecular pathways involved at the conceptus-maternal interface, especially in regard to miRNA-mRNA interactions. Using high-throughput sequencing technology, we were able to reveal a wide range of miRNAs and isomiRs expressed in the porcine endometrium during the estrous cycle and pregnancy. Although for a long time isomiRs were linked to sequencing errors [6], recently it has been shown that they can work cooperatively with canonical miRNAs to affect various biological processes [37]. In our analyses aimed at characterizing isomiRs, we focused only on template changes, whereas non-template [38] ones were neglected. The number of modifications for annotated miRNAs ranged from 0 up to 20, with the highest numbers of isomiRs found for miR-10a-5p (20 isomiRs), miR-21 (17) and miR-140-3 (14). Interestingly, read counts for some isomiRs were more abundant than for mature, canonical miRNAs (*e.g.*, ssc-miR-30a-5p, miR-30d and miR-99a). Modifications of length were related to ADD and DEL of 1 up to 6 nucleotides, and were present at 3’ or 5’ ends, or at both ends simultaneously. However, the majority of the variants produced resulted from differences in the 3´ terminus of the isomiR.

Stem-loop real-time RT-PCR was used to confirm miRNA profiles of several DE miRNAs and isomiRs found in sequencing. For example, isomiR of miR-191-5p with a G deletion on the 3’ end (miR-191-5p DEL G) demonstrated a similar expression profile to canonical miR-191-5p. The isomiR products can frequently be more abundant than the miRBase reference entry and in the case of miR-191-5p DEL G normalized read counts were 2–3 fold higher in comparison to canonical miR-191-5p. Although DEL or ADD at the 3’ terminus of miRNAs resulted in numerous length variations, the consequences of changes in the 5’ end could be more profound. This specific region bears a 6–8 nt long substring within the first 8 nt of the miRNA (usually from the 2 to 8 nt positions) that is responsible for target recognition [19]. Consequently, sequence modifications can cause a change in the seed region and affect the target repertoire of particular miRNA. On the other hand, miRNAs/5’isomiRs originating from the same transcript may share several gene targets and therefore affect their expression in more specific manner.

As demonstrated, in our *in vitro* experiment transfection of cells with either miR-140-3p or miR-140-3p 5’ DEL U isomiR or both added simultaneously significantly downregulated specific target genes as found in our *in silico* approach. However, in regard to certain targets inhibition of mRNA levels could not be observed after 24 h from transfection. This could be explained at least partially by time dependent target regulation in response to miRNA [39, 40]. Other results suggest that in the physiological conditions more complex interactions between mRNA and miRNA occurs influencing target recognition, including other context determinants outside of seed sites [41, 42]. Moreover, four types of seed-matched known to be selectively conserved [19] have following hierarchy of site efficacy: 8mer > 7mer-m8 > 7mer-A1 > 6mer [41]. Nonetheless, target prediction algorithms are still recognized as essential tools for discovery and characterization of miRNA function because experimental data is still limited. As verified by our *in vitro* experiment, available prediction tools can be useful in deciphering complex gene regulation *via* miRNA mediated mRNA inhibition, which could be crucial for events occurring during the estrous cycle and pregnancy.

Although in the present study read counts of several DE canonical miRNAs (e.g. miR-191-5p, miR-23b or miR-140-3p) were clearly lower than highly abundant miRNAs (*e.g.* miR-21, miR-143-3p or let-7-f) it seems likely that they still can sufficiently inhibit target genes, as miRNAs select targets in a dose-dependent manner [42]. Kozomara and co-workers [43] showed that even though cellular concentrations of some miRNAs differed by several orders of magnitude, yet induced similar repression of target mRNAs. Furthermore, many of DE miRNAs in the porcine endometria were co-expressed with their 3’ isomiRs and other members of family (*e.g.* 23a/b, 148a/b) harboring the same seed sequence. Thus, the cumulative concentrations of microRNA/isomiRs or miRNAs from the same family may reach a level of biological activity or even multiple the effects.

Functional annotation analysis provided insight into processes that might be affected by DE miRNAs. The top processes for PD20 *vs*. CD20 and PD16 *vs*. CD16 comparisons were linked to cellular development, cell cycle, inflammatory response, cell morphology or embryonic development, indicating the involvement of miRNAs in tissue remodeling and preparation of the uterus for subsequent embryo implantation and placentation. Among the molecules involved in these processes were miR-205-5p that could affect VEGF expression [44] or have an impact on the regulation of the epithelial-mesenchymal transition by targeting the E-cadherin repressors ZEB1 and ZEB2 [45, 46], and miR-34a-5p that regulates apoptosis through the SIRT1-p53 pathway [47].

Even though implantation in pigs is noninvasive, proteolytic activity of porcine embryos *in vitro* has been demonstrated [48]. Moreover, culture medium conditioned by porcine embryos exhibited proteolytic activity that operated under acidic conditions [49]. Physiological reduction of embryo invasiveness in pigs is accomplished *via* different protease inhibitors and a thick glycocalyx present on the surface of the endometrium [50, 51]. In contrast, reduced expression of anti-adhesive molecules such as mucins on the endometrial surface is also required for embryo implantation [52]. Here, we observed decreased expression of miR-203 in endometrium from D16 pregnant pigs in comparison to D12, while on D20 the level started to increase. Interestingly, miR-203 was found to inhibit invasiveness of cancer cells, reducing their migration *via* down-regulation of LIM and SH3 domain protein 1 (*LASP1*, [53]). Moreover, Bhat-Nakshatri et al. [23] demonstrated that expression of several miRNAs, including miR-203, is up-regulated in MCF-7 breast cancer cell lines after estrogen treatment. Estrogen-dependent regulation of miR-203 levels in the porcine endometrium would be speculative; however, the profile of miR-203 expression observed in the current study corresponds to the biphasic synthesis of estrogen by porcine embryos [28, 29].

Molecules synthesized by both embryos and the uterus may act locally in an autocrine, juxtacrine or paracrine manner or affect gene expression by distal tissues *via* endocrine pathways. An increasing body of evidence indicates that exosomes/microvesicles released by different cell types contain miRNAs, which provide an alternative mode of cell-to-cell communication [54]. Our recent study demonstrated that miR-125b, highly expressed in porcine conceptuses, can also be found in extracellular vesicles isolated from uterine flushings and is likely to regulate gene expression in endometrial epithelial cells [22]. A recent study by Zhu et al. [55] demonstrated that miR-23b by targeting TGFβ-activated kinase 1/MAP3K7 binding protein 2 (TAB2), TAB3 and an inhibitor of NF-κB kinase subunit α (IKK-α), suppresses activation of nuclear factor κ-B (NF-κB) and inflammatory cytokine expression induced by IL17, tumor necrosis factor α (TNFα) or IL1β. Interestingly, rapid embryonic growth between D11-D12 in pigs is associated with increased IL1β synthesis, while on subsequent days its expression decreases and uterine IL1β content declines on D15, reaching a nadir by D18 of pregnancy [56]. This cytokine not only stimulates embryonic growth or cell adhesion but also increases the activity of phospholipase A2 [57], which is responsible for release of arachidonic acid from cell membranes, used for prostaglandin synthesis. Interestingly, lower levels of miR-23b were observed on D12 in comparison to D16 in pregnant endometrium.

Although pigs exhibit true epitheliochorial placentation that is distinct from that observed in humans or rodents [58], blood vessel formation is equally imperative in all mammals to create a proper environment for exchange of nutrients, gases and metabolites between conceptuses and the endometrium. This process is tightly controlled by angiogenic factors, among which the most prominent is VEGF [59]. In the present study, an altered expression profile of miR-205 was observed during pregnancy, with decreased levels on D20. In the porcine endometrium, increased VEGF expression was observed during pregnancy on D16 and D20 [60], concomitant with intense blood vessel formation in the newly forming placenta [61]. Wu et al. [44] have already shown that miR-205 may directly affect expression of VEGF, through recognition of its 3’UTR region. Under physiological conditions, angiogenesis is strictly regulated *via* actions of pro- and anti-angiogenic factors [62] and a balance in their expression levels must be maintained for proper development of blood vessels in many tissues, including the endometrium.

Recently, two independent studies in pigs [12, 13] demonstrated miRNA expression in the porcine endometrium and implicated it in the regulation of embryo implantation or fetal loss. Nonetheless, we emphasize that our approach provided new knowledge about miRNA expression in the porcine endometrium, since deep sequencing analysis revealed a large repertoire of miRNAs and isomiRs during the estrous cycle and early pregnancy. Wessels at al. [12] showed that levels of 13 miRNAs were increased and 16 decreased in non-pregnant (cyclic endometria collected at mid-estrus) *vs.* day 20 healthy endometria. Some of the identified miRNAs, miR-27a, miR-30d, miR-205 and miR-574, were also present in our dataset with similar expression profiles. Su et al. [13] analyzed miRNA expression in endometrial samples but unfortunately they were collected on days 15, 26 and 50 of pregnancy, which precludes comparison with our dataset.

## Conclusions

A comprehensive catalog of transcriptome changes related to miRNA synthesis occurring during the time of maternal recognition of pregnancy and conceptus attachment in pigs was revealed. Bioinformatic analysis showed that microRNAs via affecting number of pathways and specific processes such those related to cell-to-cell communication, cellular development, cell growth and proliferation might be involved in early pregnancy events, *e.g.* embryo implantation and placentation. Moreover our *in vitro* approach proved that isomiRs can be as much important factors regulating gene expression as canonical miRNAs in porcine endometrial cells during pregnancy. Nevertheless, many questions remained unanswered, including why such a big repertoire of isomiRs exist in the porcine endometrium, how genes related to miRNA synthesis could be responsible for generation of such a diversity in these small non-coding RNAs, how changes in miRNA/isomiR content may affect female fertility? Regardless of whether regulation of embryo-maternal interactions *via* miRNA/isomiR action is crucial or unique mechanism during early pregnancy, identification of miRNAs and their modified counterparts in conjunction with indication of their possible role can advance our understanding of the mechanism underlying maternal recognition of pregnancy and embryo implantation in mammals.

## Methods

### Animals

Hampshire x Duroc crossbred gilts at the same age (7–8 months) and genetic background from one commercial herd were inseminated at 12 and 24 h after detection of third estrus. Samples were collected *post mortem* on day (D) 10 (*n* = 8–9), 12 (*n* = 7), 16 (*n* = 8–9) and 20 (*n* = 7–8) of estrous cycle and pregnancy. Until D16 of gestation uterine horns were flushed twice with 20 ml 0.01 M PBS (pH 7.4) to remove conceptuses. Uterine horns from all analyzed periods were opened longitudinally, opposite to the mesometrium side, endometrium were cut into slices and immediately snap frozen in liquid nitrogen and stored at −80 °C for further analysis or fixed in 2 % paraformaldehyde for immunostaining. On D20 of pregnancy, endometrial samples were collected directly from implantation sites after embryos with trophoblast being removed. The day of pregnancy was confirmed by the size and morphology of conceptuses as follows: D10 (spherical/ovoid; diameter 2–8 mm), D12 (filamentous; >100 mm long), D16 (elongated), and D20 (trophoblast tissue and embryos with evident vascularization). All experiments were conducted in accordance with the International Guiding Principles for Biomedical Research Involving Animals and were approved by the Local Research Ethics Committee (approval No 25/2010).

### Real-time RT-PCR quantitation

For Sybr Green-based gene expression, assays were carried out according to the protocol developed previously [22]. Gene specific primers for real-time RT-PCR reactions are listed in Additional file 11: Table S7.

### Immunolocalization of DICER1 and AGO2 proteins in the porcine endometria

Paraformaldehyde fixed, paraffin embedded uterine samples were cut into 5 μm thick sections and were mounted on chromogelatin-precoated slides (Superfrost plus, Menzel-Gläser, Braunschweig, Germany). After paraffin removal, tissue was dehydrated in an ethyl alcohol grades (98–50 %). Non-specific antigens were blocked using 10 % donkey serum (Jackson ImmunoResearch Laboratories, Inc., West Grove, PA, USA) in PAV (0.1 M PBS 0.1 % BSA, 0.05 % timerosal). Incubation with primary antibodies, monoclonal mouse anti-DICER1 (1:150, Abcam) or polyclonal rabbit anti-AGO2 (1:50; Abcam) was performed overnight at 4 °C. Afterwards, slides were washed in TBS buffer (50 mM Tris-HCl, pH 7.4; 150 mM NaCl) and incubated for 1 h with secondary antibodies (Life Technologies, Inc., Carlsbad, CA, USA): donkey anti-rabbit IgG conjugated with Alexa Fluor 488 (1:5 000) or donkey anti-mouse conjugated with Alexa Fluor 594 (1:5 000). Negative control sections were stained with primary antibodies replaced with either 10 % normal rabbit serum (NRS, Abcam) or mouse IgG_1_ (1:150; Abcam). Finally, sections were mounted in UltraCruz Mounting Medium (Santa Cruz Biotechnology) containing 4',6-Diamidino-2-Phenylindole (DAPI). Sections were examined under fluorescent microscope Axio Imager Z1 (Carl Zeiss, Germany) equipped with AxiCam MRM. Images were analyzed with AxioVision v. 4.8 (Carl Zeiss).

### Small RNA library preparation and Illumina sequencing

Total RNA isolated from endometria of pregnant (P) and cyclic (C) animals from day 12 (*n* = 5 for P; *n* = 4 for C), D16 (*n* = 5 for both states) and D20 (*n* = 6 for P; *n* = 5 for C) were used for small RNA libraries preparation. In brief, 5 μg of total RNA form each sample was used for library preparation with NEXTflex Small RNA Sequencing Kit (BiooScientific, Austin, TX, USA). All procedures were followed as stated in the supplier protocol. 3’ and 5’ adapters were ligated to the RNA using AIR ligase and T4 RNA ligase, respectively. During the 5’ ligation, NEXTflex RT primers were also incorporated to the RNA. After 3’ and 5’ primer ligation, the ligation products were purified by Clean & Concentrator 5 Kit (Zymo Research, Irvine, CA, USA) to eliminate unligated products. PCR amplification of the ligated products was accomplished by using primer barcodes 1–12 (NEXTflex Small RNA Barcode Primers, BiooScientific) and DuroTaq 5x PCR Master Mix (BiooScientific). Concentration of each library was determined with Agilent High Sensitivity DNA Kit using the Agilent 2100 Bioanalyzer (Agilent Technologies, Waldbronn, Germany). Final concentration of 30 nmol/l of each library was used to prepare 3 pools for multiplex analysis. Size fractioning of the pooled cDNA was done by 2 % agarose gel electrophoresis in the presence of 0.5 ng/μl of ethidium bromide. Small RNA fraction with ligated adaptors ranging around 140–160 bp (Additional file 12: Figure S5) was excised from the gel with X-TRACTA II (Biozym Scientific GmbH, Hessisch Oldendorf, Germany), purified (Ultrafree-DA, Millipore, Billerica, MA, USA) and sequenced using TruSeq SR cluster Kit v.2 and Genome Analyzer GAIIx (Illumina, Inc., San Diego, CA, USA) according to the vendor’s recommended protocol for small RNA sequencing-by-synthesis technology. NGS experiments, described according to MIAME guidelines, have been deposited in NCBI’s Gene Expression Omnibus (GEO, http://www.ncbi.nlm.nih.gov/geo) repository with accession numbers GSE64863, SRP052027.

### Data pre-processing, miRNA annotation and statistical analysis

The Illumina Sequencing Analysis Viewer v. 1.8.4 was used for quality control of sequencing. Information encoded in *. TIF files was converted to numerical data and saved as FASTQ files, which were further subjected to the analysis using a locally installed Galaxy platform [63]. Schematic illustration of the pipeline used for data pre-processing with detailed description is presented in Additional file 13: Figure S6. Sequences with at least 10 reads/per sample, in at least 4 samples (except group CD12, where 3 out of 4 samples were considered) for at least one of the analyzed groups were used for annotation. BLAST (*Basic Local Alignment Search Tool*) tool within Galaxy was used in order to annotate sequence tags to porcine and mammalian mature and stem-loop miRNAs present in miRBase v. 18 [16], NCBI [64], EMBL [65], Ensembl [66]. miRNA sequences, showing 100 % identity with the reference sequence were used for normalization and statistical analysis in EdgeR v. 3.0.8 for BioconductorR [18]. ANOVA-like analysis was used for comparisons between days of the same reproductive status and moderated t-test for comparative analysis between corresponding days of the estrous cycle and pregnancy. Differentially expressed miRNAs were found by applying log2 fold change cutoff 1 and threshold *p* < 0.05 corrected with Benjamini-Hochberg false discovery rate (FDR). Before preparation of the final manuscript annotated sequences were updated to the miRBase v. 20.

### miRNA functional analysis and target prediction

Unsupervised hierarchical clustering (HCL) and Self Organizing Tree Algorithm (SOTA) were performed in the MeV software [67] using mean centered values to evaluate gene expression patterns. For signaling pathways and molecular functions IPA [68] tool was used. For statistical significance the right-tailed Fisher’s exact test using a threshold *p*-value < 0.05 after application of Benjamini-Hochberg method of multiple testing correction was applied. The network analysis and biological functions were performed in IPA using separate gene list for each comparison. Target prediction for DE miRNAs was done using IPA and miRWalk [20]. IPA annotations are based on the human, mouse and rat databases. Identical miRBase gene names across species indicate orthologs, therefore in order to upload our data to IPA human identifiers for all miRNAs were used.

### miRNA quantitation by stem-loop RT-PCR

Four canonical miRNAs (miR-1, miR-23b, miR-191-5p and miR-205), three isomiRs (miR-191-5p DEL G, miR-23a DEL C, miR-30a-3p DEL C) and two miRNAs not known in pig (miR-203 and miR-302), which showed differential expression in Next Generation Sequencing (NGS) were selected for RT-PCR validation using TaqMan MicroRNA Assays (Life Technologies, Additional file 14: Table S8). miR-148a, miR-199b and let-7d-3p were used as a reference miRNAs, selected on the basis of constant average expression in endometria of all groups found by NGS. Briefly, 10 ng of total RNA was reverse transcribed using MultiScribe™ Reverse Transcriptase and RT primers, added separately for each miRNA according to the supplier’s instructions. Real-time PCR was performed in a final volume of 10 μl using 0.7 μl of RT product, 0.5 μl of specific primers with probes and TaqMan Universal PCR Master Mix II (Life Technologies). Amplification was performed with initial denaturation for 10 min at 95 °C, followed by 45 cycles of 15 sec at 95 °C and 60 sec at 60 °C on an ABI HT7900 sequence detection system (Life Technologies). PCR reactions were performed in duplicate, and negative controls, prepared either by replacing the cDNA template with water or without addition of the reverse transcriptase, were amplified in each run.

### Primary cell culture

Porcine stromal cells were isolated from endometria collected on D16 of pregnancy (*n* = 6) as described previously with some modifications [69]. Briefly, uterine horns were washed with sterile phosphate-buffered saline (PBS, pH 7.4) to prove pregnancy by the appearance of elongated conceptuses. Endometrial tissue was separated from the myometrium and digested with 0.2 % (w/v) dispase in Hanks’ balanced salt solution (Sigma-Aldrich) at room temperature for 50 min. Remaining endometrial tissue was minced with scissors, placed in 0.06 % collagenase in M199 containing 1 % bovine serum albumin (BSA) and digested for 80 min at 37 °C. Cellular viability, determined by trypan blue dye exclusion was approximately 95 %. Cells were plated in 6-well culture plates at a density of 5 x 10^5^ cells/well in culture medium (M199; Sigma-Aldrich) and incubated at 37 °C in a humidified atmosphere of 95 % air:5 % CO2. After 24 h of seeding, stromal cells were washed gently with PBS to remove contaminating epithelial cells. Afterwards, cells were cultured for additional 24 h to complete cell adhesion before initiation of the experiment, until approximately 60 % of confluence.

### Transfection of primary cells

Canonical porcine miR-140-3p (MC11183) and/or porcine isomiR miR-140-3p 5’ DEL U (MC25293) mimics as well as miRNA mimic negative control (4464061) (Life Technologies) were suspended in M199 medium without supplements. Cells washed with PBS were transfected using Lipofectamine RNAiMAX reagent (Life Technologies) with control (50 nM), canonical miR-140-3p (50 nM), miR-140-3p isomiR (50 nM) or added simultaneously miR-140-3p and isomiR (25 nM each) and incubated for 12 h. Afterward, cells were washed with PBS and incubated for an additional 24 h in fresh M199 medium supplemented with 10 % NCS and antibiotics. Each treatment was performed in duplicate. After treatment cells were harvested and total RNA was isolated with the miRVana miRNA isolation kit (Life Technologies). Expression levels of activin A receptor, type IIB (*ACVR2B*), potassium channel, calcium activated large conductance subfamily M alpha, member 1 (*KCNMA1*), sirtuin 1 (*SIRT1*), leukemia inhibitory factor (*LIF*) and lysophosphatidic acid receptor 2 (*LPAR2*), as well as those of the two reference genes *ACTB* and hypoxanthine phosphoribosyltransferase 1 (*HPRT1*), were assessed using gene expression assays (Additional file 15: Table S9) and One-Step RT-PCR Master Mix reagent kit (Life Technologies). Two-step real-time RT-PCR was performed to determine miR-140-3p (005800_mat, Life Technologies) and its isomiR (471823_mat, Life Technologies), as well as let-7d -3p (reference gene, Additional file 14: Table S8) expression by using microRNA assays (TaqMan) as described previously.

### Statistical analysis of RT-PCR results

Raw data of the fluorescence values were imported from the SDS 2.3 software into PCR Miner to calculate efficiency [70]. Subsequently NormFinder [71] was used to select the most stable reference gene. Relative expression levels of genes of interest were normalized to geometric mean [72] of *ACTB* and *PPIB* gene expression or in case of miRNAs relatively to let-7d-3p. The normal distribution was tested by the Kolmogorov-Smirnov test with Dallal-Wilkinson-Lillie approximation. Statistical analyses were performed using two-way ANOVA followed by Bonferroni post-hoc test (GraphPad Prism 5.0; GraphPad Software Inc., San Diego, CA, USA). For *in vitro* study, relative expression levels of genes of interest were normalized to geometric mean of *HPRT1* and *ACTB*, whereas let-7d-3p was used as reference for miR-140-3p and isomiR miR-140-3p 5’ DEL U. Statistical analyses were performed using paired one-way ANOVA (GraphPad Prism 5.0). Differences were considered significant at *p* < 0.05.

## Additional files

Additional file 1: Table S1. Average values representing read counts after subsequent steps of pre-analysis of raw data obtained from Illumina sequencing. Letters indicate pregnancy (P) and the estrous cycle (C), whereas numbers a day (*n* = 5 for all groups, except P12 [*n* = 4] and P20 [*n* = 6]). Valid sequences were obtained by subtracting number of reads with length <17 nt and reads with unknown bases from reads with found adaptor sequences. (DOCX 17 kb) Additional file 2: Figure S1. General characteristic of the reads obtained from Illumina sequencing. (A) Distribution of sequences lengths over all sequences. X-axis represents sequence length and y-axis number of reads of small RNAs. (B) Quality scores across all bases of the sequenced tags. Phred quality score is represented on y-axis and score distribution depending on the base pair (bp) position in sequence on x-axis. (C) GC distribution over all sequences in library. Mean GC content represented as % of all nucleotides is plotted on x-axis and read count on y-axis. (TIF 1927 kb) Additional file 3: Table S2. List of sequences annotated and not annotated to known porcine and mammalian miRNAs. In total, 873 sequences, showing 100 % homology to the reference sequence, were annotated to mature or stem-loop miRNAs represented in miRBase v. 18, Ensembl, EMBL or NCBI databases. (XLSX 781 kb) Additional file 4: Figure S2. Pre-miRNA sequences with assigned miRNAs/isomiRs not included in miRNA characterization. Reference/canonical miRNA found in miRBase, corresponding to specific pre-miRNA was marked in bold and underlined. All sequences found in our dataset were assigned to specific miRNA transcript and ambiguous sequences were underlined. (PDF 77 kb) Additional file 5: Table S3. Lists of DE miRNAs found in pregnant and cyclic endometria on different days of the estrous cycle and early pregnancy. Differences were considered significant with FDR < 0.05 and −1 < log FC > 1. Comparison analysis between different days of the estrous cycle or pregnancy performed in EdgeR using ANOVA-like analysis. Expression profiles between pregnancy and estrus cycle were compared using t-test. Values in red correspond to up-regulated and in green to down-regulated sequences in selected comparison. Prefixes stand for: hsa - Homo sapiens, mmu - Mus musculus, ssc - Sus scrofa, bta- Bos taurus, cfa - Canis familiaris, eca - Equus caballus, ggo - Gorilla gorilla, oan - Ornithorhynchus anatinus, cgr - Cricetulus griseus, mdo - Monodelphis domestica, rno - Rattus norvegicus, age - Ateles geoffroyi, lla - Lagothrix lagotricha, ptr - Pan troglodytes, sha - Sarcophilus harrisii, ppy - Pongo pygmaeus, mml - Macaca mulatta, oar - Ovis aries. (XLSX 604 kb) Additional file 6: Figure S3. miRNAs and isomiRs classified to the miR-23 (A) and miR-148 (B) families, harboring the same seed sequences. Reference/canonical miRNA found in miRBase, corresponding to specific pre-miRNA was marked in bold and underlined. Underlined sequence of miR-23a/b isomiR could not be uniquely assigned to one member of given family. (PDF 149 kb) Additional file 7: Table S4. List of DE miRNAs found in pregnant and cyclic endometria organized into 7 clusters according to the SOTA algorithm. Numbers in brackets indicate numbers of miRNAs displaying similar profiles and assigned to each cluster. (XLSX 19 kb) Additional file 8: Table S5. Results of IPA functional annotations and processes enriched with DE miRNAs between different days of the estrous cycle and early pregnancy. All mature, canonical miRNAs which were orthologs to human miRNAs (input data with human identifiers) were used for analysis. (XLSX 16 kb) Additional file 9: Table S6. Results of IPA functional annotations and processes enriched with common targets for canonical miR-140-3p (seed sequence ACCACAG) and its 5’ isomiR with seed sequence CCACAGG. (XLSX 21 kb) Additional file 10: Figure S4. Cross-species comparison of 3’UTRs of putative target genes of miR-140-3p and isomiR miR-140-3p 5’ DEL U. (PDF 95 kb) Additional file 11: Table S7. Primers, GenBank accession numbers and product lengths of miRNA synthesis/transport-related genes studied in real-time RT-PCR analysis. (DOCX 17 kb) Additional file 12: Figure S5. Size-fractioning of pooled small RNA libraries. Libraries obtained with NEXTflex™ Small RNA Sequencing Kit and NEXTflex Small RNA Barcode Primers were separated on 2 % agarose gel and visualized with ethidium bromide. Small RNAs with ligated adaptors correspond to the band of 140–160 bp. Band at around 120 bp corresponds to adaptor dimers. Bands of 140–160 bp were excised from gel, purified and used for sequencing. M – molecular mass marker; C – control purchased with NEXTflex™ Small RNA Sequencing Kit; MP1, MP2 and MP3 – pooled small RNA libraries. (TIF 664 kb) Additional file 13: Figure S6. Workflow of data pre-processing obtained by Illumina sequencing. FASTQ files generated from the sequencing were demultiplexed in order to obtain information from single library. Phred quality score filtering was used with the minimal score of 20, and the notion that all of the nucleotides followed this rule. After removing primer sequences, and file conversion into TXT format, read counts for all sequences were calculated. Finally, for further analysis only sequences that met fallowing rules were used: i) at least 10 reads per sample, ii) for at least 75 % of samples iii) in at least one of the analyzed groups. (TIF 126 kb) Additional file 14: Table S8. miRNA/isomiR names, sequences and assays ID of molecules validated by stem-loop real-time RT-PCR in the porcine endometrium. (DOCX 18 kb) Additional file 15: Table S9. Names, Assay IDs, GenBank accession numbers and product length of selected genes analyzed in real-time PCR in an *in vitro* study. (DOCX 15 kb)
