# Supplementary material for: Expression of microRNAs and isomiRs in the porcine endometrium: implications for gene regulation at the maternal-conceptus interface
Source: BMC Genomics. 2015 Nov 6;16:906. doi: 10.1186/s12864-015-2172-2 (PMC4636777; doi:10.1186/s12864-015-2172-2)
Supplement: Additional file 1: Table S1. — Average values representing read counts after subsequent steps of pre-analysis of raw data obtained from Illumina sequencing. Letters indicate pregnancy (P) and the estrous cycle (C), whereas numbers a day (n = 5 for all groups, except P12 [n = 4] and P20 [n = 6]). Valid sequences were obtained by subtracting number of reads with length <17 nt and reads with unknown bases from reads with found adaptor sequences. (DOCX 17 kb) [file 12864_2015_2172_MOESM1_ESM.docx]

**Additional file 1: Table S1.** Average values representing read counts after subsequent steps of pre-analysis of raw data obtained from Illumina sequencing. Letters indicate pregnancy (P) and the estrous cycle (C), whereas numbers a day (n=5 for all groups, except P12 [n=4] and P20 [n=6]).Valid sequences were obtained by subtracting number of reads with length <17 nt and reads with unknown bases from reads with found adaptor sequences.

| Step of pre-analysis | Day | | | | | |
| --- | --- | --- | --- | --- | --- | --- |
|  |  |  |  |  |  |  |
|  | PD12 | PD16 | PD20 | CD12 | CD16 | CD20 |
| Raw sequences | 8192801 | 6603153 | 9200516 | 8781024 | 7805662 | 6605189 |
| Reads with phred quality score >20 | 3979491 | 3269835 | 4611156 | 4258811 | 3928705 | 3210430 |
| Reads with adaptor sequences | 3372147 | 2817921 | 3764808 | 3660779 | 3358081 | 2682578 |
| Reads with length <17nt | 13424 | 4843 | 6009 | 5186 | 4760 | 4428 |
| Reads with unknown bases | 48516 | 38512 | 52030 | 58596 | 47896 | 37553 |
| Valid sequences | 3310207 | 2774566 | 370679 | 3596997 | 3305425 | 2640597 |
| Unique sequences | 57492 | 47416 | 59024 | 46748 | 54437 | 51868 |
