# Supplementary figures and images for: Expression of microRNAs and isomiRs in the porcine endometrium: implications for gene regulation at the maternal-conceptus interface
Source: BMC Genomics. 2015 Nov 6;16:906. doi: 10.1186/s12864-015-2172-2 (PMC4636777; doi:10.1186/s12864-015-2172-2)

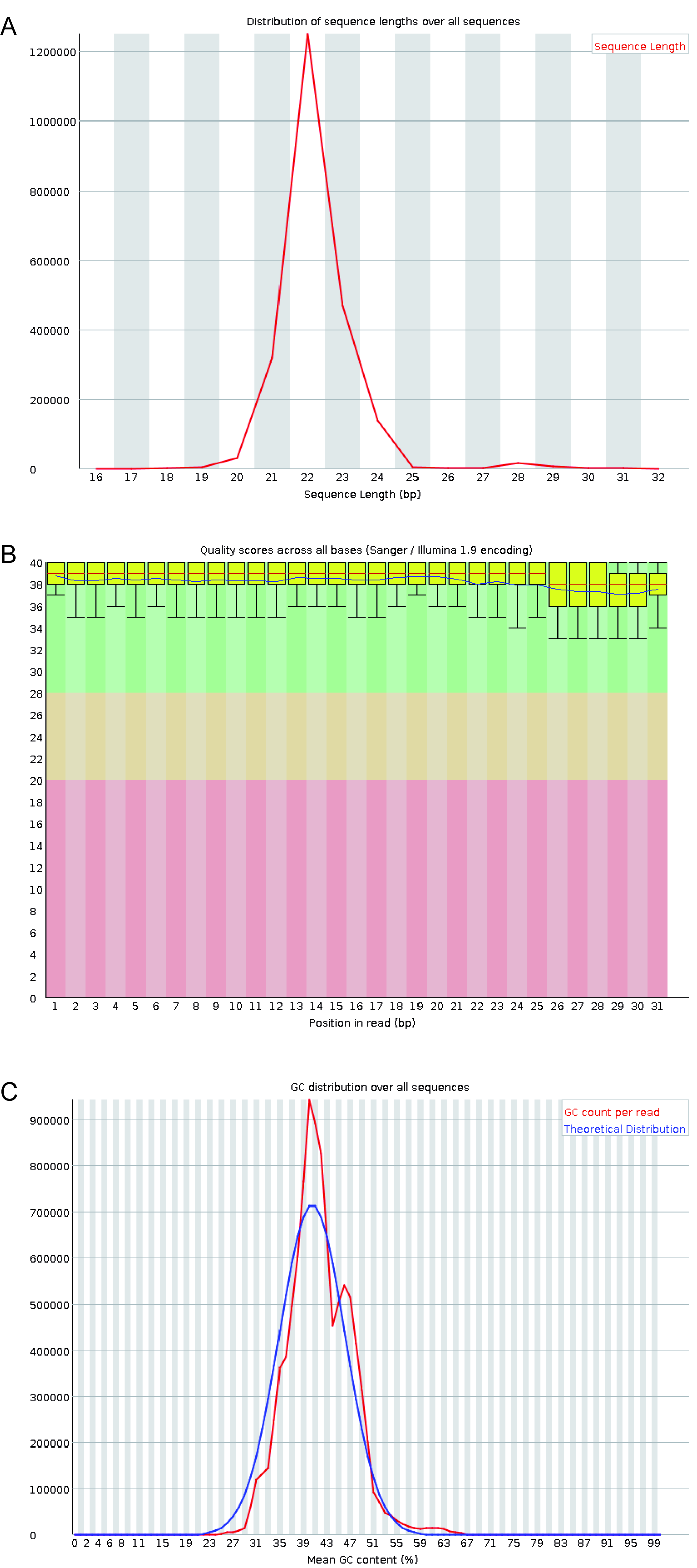

Supplement: Additional file 2: Figure S1. — General characteristic of the reads obtained from Illumina sequencing. (A) Distribution of sequences lengths over all sequences. X-axis represents sequence length and y-axis number of reads of small RNAs. (B) Quality scores across all bases of the sequenced tags. Phred quality score is represented on y-axis and score distribution depending on the base pair (bp) position in sequence on x-axis. (C) GC distribution over all sequences in library. Mean GC content represented as % of all nucleotides is plotted on x-axis and read count on y-axis. (TIF 1927 kb) [file 12864_2015_2172_MOESM2_ESM.tif]

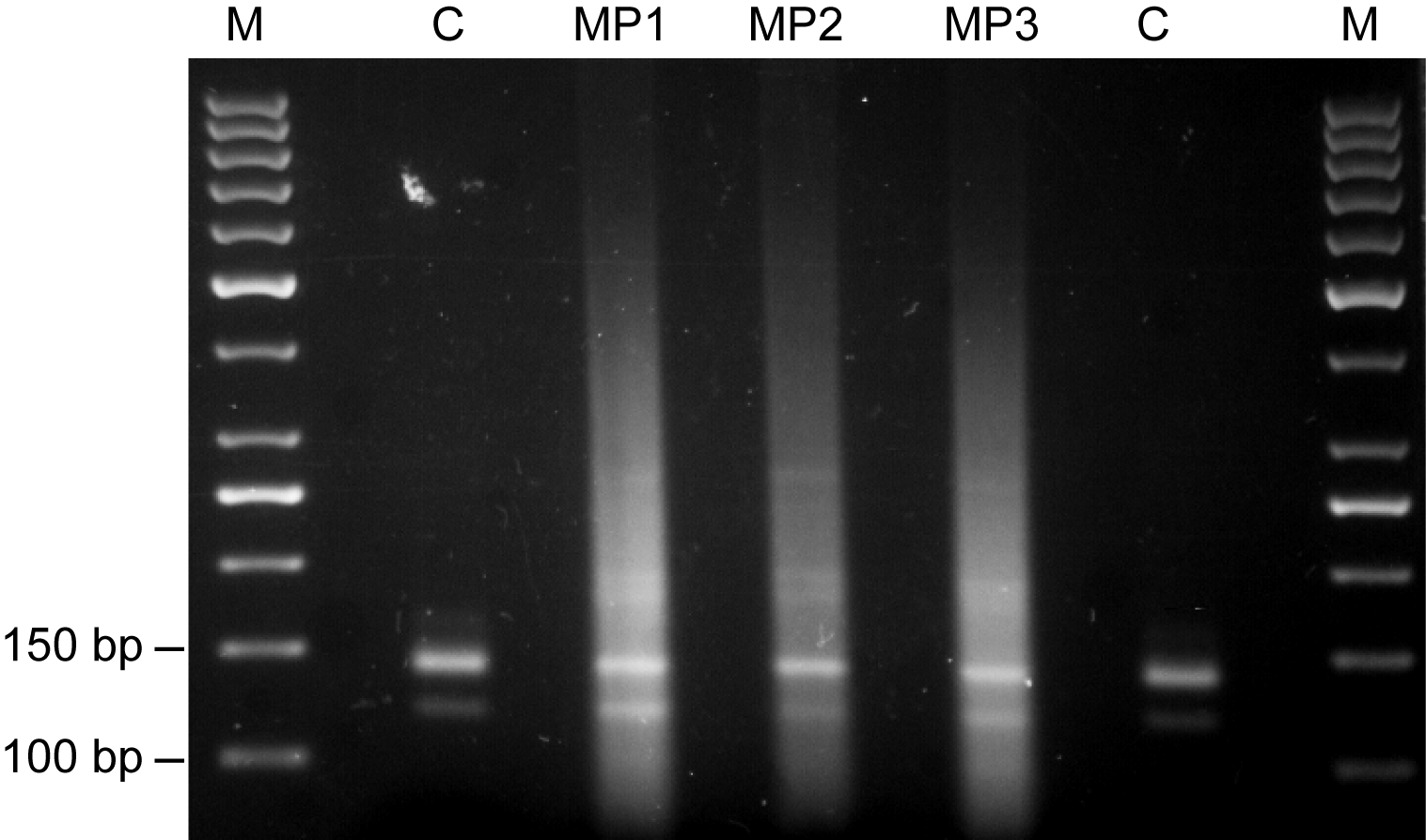

Supplement: Additional file 12: Figure S5. — Size-fractioning of pooled small RNA libraries. Libraries obtained with NEXTflex™ Small RNA Sequencing Kit and NEXTflex Small RNA Barcode Primers were separated on 2 % agarose gel and visualized with ethidium bromide. Small RNAs with ligated adaptors correspond to the band of 140–160 bp. Band at around 120 bp corresponds to adaptor dimers. Bands of 140–160 bp were excised from gel, purified and used for sequencing. M – molecular mass marker; C – control purchased with NEXTflex™ Small RNA Sequencing Kit; MP1, MP2 and MP3 – pooled small RNA libraries. (TIF 664 kb) [file 12864_2015_2172_MOESM12_ESM.tif]

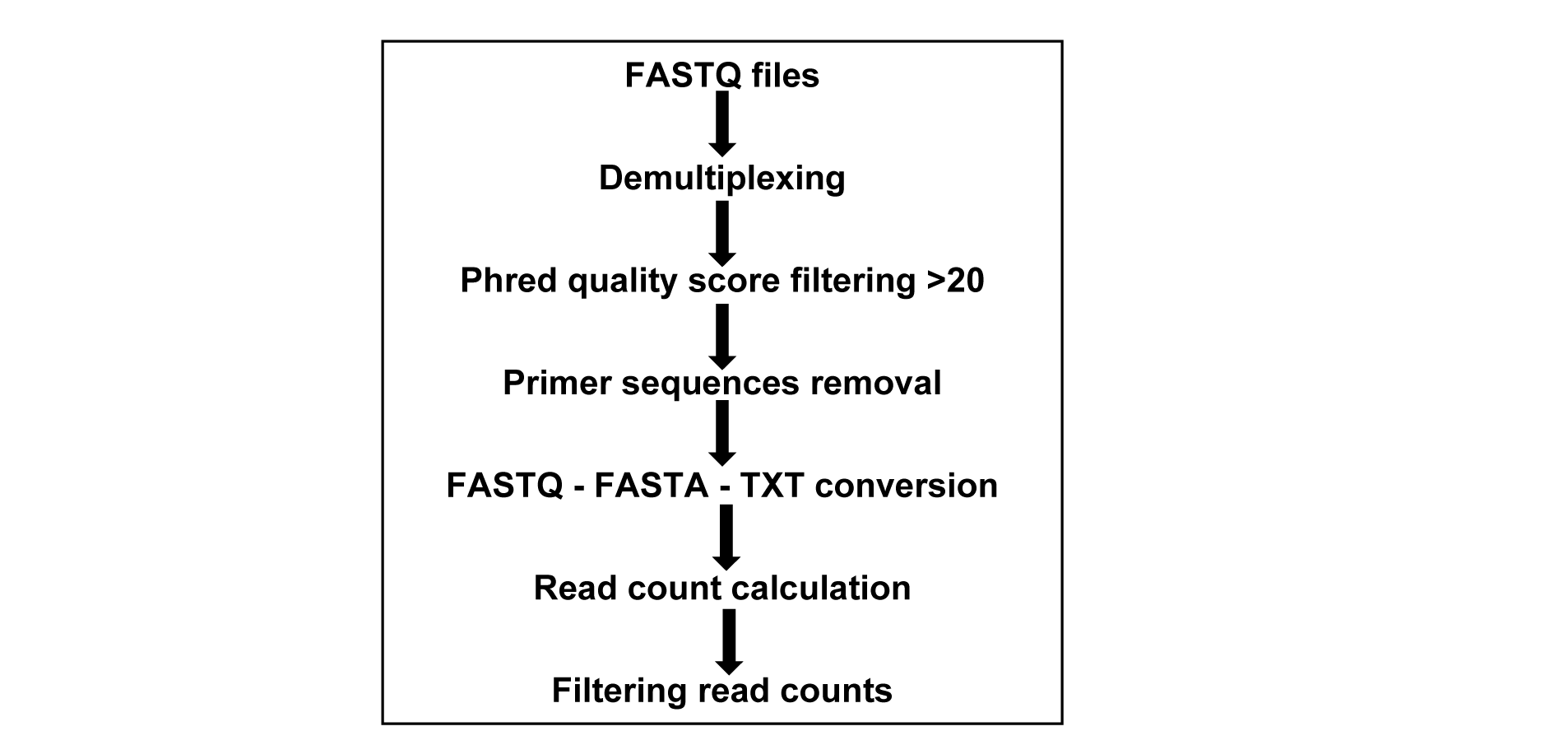

Supplement: Additional file 13: Figure S6. — Workflow of data pre-processing obtained by Illumina sequencing. FASTQ files generated from the sequencing were demultiplexed in order to obtain information from single library. Phred quality score filtering was used with the minimal score of 20, and the notion that all of the nucleotides followed this rule. After removing primer sequences, and file conversion into TXT format, read counts for all sequences were calculated. Finally, for further analysis only sequences that met fallowing rules were used: i) at least 10 reads per sample, ii) for at least 75 % of samples iii) in at least one of the analyzed groups. (TIF 126 kb) [file 12864_2015_2172_MOESM13_ESM.tif]
