# Supplementary material for: Expression of microRNAs and isomiRs in the porcine endometrium: implications for gene regulation at the maternal-conceptus interface
Source: BMC Genomics. 2015 Nov 6;16:906. doi: 10.1186/s12864-015-2172-2 (PMC4636777; doi:10.1186/s12864-015-2172-2)
Supplement: Additional file 4: Figure S2. — Pre-miRNA sequences with assigned miRNAs/isomiRs not included in miRNA characterization. Reference/canonical miRNA found in miRBase, corresponding to specific pre-miRNA was marked in bold and underlined. All sequences found in our dataset were assigned to specific miRNA transcript and ambiguous sequences were underlined. (PDF 77 kb) [file 12864_2015_2172_MOESM4_ESM.pdf]

A

Reference miR-9-1

Pre-miR-9-1 CGGGGUUGGUUGUUA**UCUUUGGUUAUCUAGCUGUAUGA**GUGGUGUGGAGUCUUCAUAAAGCUAGAUAACCGAAAGUAAAAAUAACCCCA

UCUUUGGUUAUCUAGCUGUAUG

UCUUUGGUUAUCUAGCUGUAU

UCUUUGGUUAUCUAGCUGUA

Reference miR-9-2

Pre-miR-9-2 GGAAGCGAGUUGUUA**UCUUUGGUUAUCUAGCUGUAUGA**GUGUAUUGGUCUUCAUAAAGCUAGAUAACCGAAAGUAAAAACUCCUUCA

UCUUUGGUUAUCUAGCUGUAUG

UCUUUGGUUAUCUAGCUGUAU

UCUUUGGUUAUCUAGCUGUA

Reference miR-9

Pre-mir-9-3 CCCGUUUCUC**UCUUUGGUUAUCUAGCUGUAUG**AGUGCCACAGAGCCGUCAUAAAGCUAGAUAACCGAAAGUAGAAAUGACU

UCUUUGGUUAUCUAGCUGUAUG

UCUUUGGUUAUCUAGCUGUAU

UCUUUGGUUAUCUAGCUGUA

B

Reference miR-450a

Pre-miR-450a GUCUGUCAAAGAAAGAUGCUAAACUGG**UUUUGCGAUGUGUUCCUAAUAU**GCAGUAUAAAUAUAUUGGGAGCAUUUUGCAUGCAUGGUUUUGUAUCACUAUACAGAU

UUUUGCGAUGUGUUCCUAAUAU

UUUUGCGAUGUGUUCCUAAUA

UUUUGCGAUGUGUUCCUAAU

Reference miR-450c-5p

Pre-miR-450c GGCACUAAACUAU**UUUUGCGAUGUGUUCCUAAUAC**GUAAUCUAAGUGUAUUGGGAACAUUUUGCAUUCGUAGUUUUGUGUC

UUUUGCGAUGUGUUCCUAAUAC

UUUUGCGAUGUGUUCCUAAUA

UUUUGCGAUGUGUUCCUAAU

C

Reference let-7a

Pre-let-7a CCCAGGU**UGAGGUAGUAGGUUGUAUAGUU**UAGAAUUGUAUCAAGGGAGAUAACUGUACAGCCUCCUAGCUUUCCUUGGGU

UGAGGUAGUAGGUUGUAUAGUUUU
UGAGGUAGUAGGUUGUAUAGUUU
UGAGGUAGUAGGUUGUAUAGUU
UGAGGUAGUAGGUUGUAUAGU
UGAGGUAGUAGGUUGUAUAG
UGAGGUAGUAGGUUGUAUA
UGAGGUAGUAGGUUGUAU

Reference let-7c

Pre-let-7c UGUGUGCAUCCGGGU**UGAGGUAGUAGGUUGUAUGGUU**UAGAGUUACACCGUGGGAGUUAACUGUACAACCUUCUAGCUUUCCUUGGAGCACACU

UGAGGUAGUAGGUUGUAUGGUUU
UGAGGUAGUAGGUUGUAUGGUU
UGAGGUAGUAGGUUGUAUGGU
UGAGGUAGUAGGUUGUAUGG
UGAGGUAGUAGGUUGUAUG
UGAGGUAGUAGGUUGUAU

D

Reference miR-103

Pre-miR-103 CUUACUGCCCUCGGCUUCUUUACAGUGCUGCCUUGUUGCAUAUGGAUCA**AGCAGCAUUGUACAGGGCUAUGA**AGGCACUGAG

AGCAGCAUUGUACAGGGCUAUGAAA
AGCAGCAUUGUACAGGGCUAUGAA
AGCAGCAUUGUACAGGGCUAUGA
AGCAGCAUUGUACAGGGCUAUG
AGCAGCAUUGUACAGGGCUAU
CAGCAUUGUACAGGGCUAUGA

Reference miR-107

Pre-miR-107 UUCUCUCUGCUUUCAGCUUCUUUACAGUGUUGCCUUGUGGCAUGGAGUUCA**AGCAGCAUUGUACAGGGCUAUCA**AAGCACAGAGAGC

AGCAGCAUUGUACAGGGCUAUCA
AGCAGCAUUGUACAGGGCUAUC
AGCAGCAUUGUACAGGGCUAU

E

Reference miR-23a-3p

Pre-miR-23a CGGCUGGGGUUCCUGGGGAUGGGAUUUGCUGCCUGUCACAA**AUCACAUUGCCAGGGAUUUCC**AAUCGACC

AUCACAUUGCCAGGGAUUUCCAAU
AUCACAUUGCCAGGGAUUUCCAA
AUCACAUUGCCAGGGAUUUCCA
AUCACAUUGCCAGGGAUUUCC
AUCACAUUGCCAGGGAUUUC
AUCACAUUGCCAGGGAUUU
AUCACAUUGCCAGGGAUU

Reference miR-23b-3p

Pre-miR-23b CUCUGGCUGCUUGGGUUCCUGGCAUGCUGAUUUGUGACUUAAGAUUAAA**AUCACAUUGCCAGGGAUUACCA**CGCAGCCAC

AUCACAUUGCCAGGGAUUACCAC
AUCACAUUGCCAGGGAUUACCA
AUCACAUUGCCAGGGAUUACC
AUCACAUUGCCAGGGAUUAC
AUCACAUUGCCAGGGAUU

F

Reference miR-27a

Pre-miR-27a UGGCCUGGGGAGCAGGGCUUAGCUGCUUGUGAGCAGGUCCACAGCAAGUCGUG**UUCACAGUGGCUAAGUUCCGC**CCCCUGGA

UUCACAGUGGCUAAGUUCCGCC
UUCACAGUGGCUAAGUUCCGC
UUCACAGUGGCUAAGUUCCG
UUCACAGUGGCUAAGUUCC
UUCACAGUGGCUAAGUUC

Reference miR-27b

Pre-miR-27b ACGAGGUGCAGAGCUUAGCUGAUUGGUGAACAGUGACUGGUUUCCGCUUUG**UUCACAGUGGCUAAGUUCUGC**ACCUGAAG

GUUCACAGUGGCUAAGUUCUGC
UUCACAGUGGCUAAGUUCUGCAC
UUCACAGUGGCUAAGUUCUGCA
UUCACAGUGGCUAAGUUCUGC
UUCACAGUGGCUAAGUUCUG
UUCACAGUGGCUAAGUUCU
UUCACAGUGGCUAAGUUC

G

Pre-mir-339-1 — Reference miR-339-5p

GCAGCCGCCG**UCCCUGUCCUCCAGGAGCUCAC**UUACCUUGGGCCGUGAGCUCCUCGAGGCCAGAGCCCGUGUCUGCCUCU

UCCCUGUCCUCCAGGAGCUCACU
UCCCUGUCCUCCAGGAGCUCAC
UCCCUGUCCUCCAGGAGCUCA

Pre-mir-339-2 — Reference miR-339

UAAGACAAGG**UCCCUGUCCUCCAGGAGCUCA**UGGUCCAAUAGAAGCAUAUAUACAAAUGGCUCUGAGGGGAGAUCCCGCUGUGGCU

UCCCUGUCCUCCAGGAGCUCAU
UCCCUGUCCUCCAGGAGCUCA

H

Pre-miR-199a-2 — Reference miR-199a-3p

CCCCGCCAACCCAGUGUUCAGACUACCUGUUCAGGGGGCUCUGAAUGUG**UACAGUAGUCUGCACAUUGGUUA**GGCUGGGC

UACAGUAGUCUGCACAUUGGUUA
UACAGUAGUCUGCACAUUGGUU
UACAGUAGUCUGCACAUUGGU
ACAGUAGUCUGCACAUUGGUUA
ACAGUAGUCUGCACAUUGGUU
ACAGUAGUCUGCACAUUGGU
CAGUAGUCUGCACAUUGGUUA
CAGUAGUCUGCACAUUGGUU

Pre-mir-199b — Reference miR-199b-3p

CCGUCUACCCAGUGUUUAGACUAUCUGUUCAGGACUCCCAAAUUG**UACAGUAGUCUGCACAUUGGUU**AGGCUGG

UACAGUAGUCUGCACAUUGGUUA
UACAGUAGUCUGCACAUUGGUU
UACAGUAGUCUGCACAUUGGU
ACAGUAGUCUGCACAUUGGUUA
ACAGUAGUCUGCACAUUGGUU
ACAGUAGUCUGCACAUUGGU
CAGUAGUCUGCACAUUGGUUA
CAGUAGUCUGCACAUUGGUU
