# Supplementary material for: Expression of microRNAs and isomiRs in the porcine endometrium: implications for gene regulation at the maternal-conceptus interface
Source: BMC Genomics. 2015 Nov 6;16:906. doi: 10.1186/s12864-015-2172-2 (PMC4636777; doi:10.1186/s12864-015-2172-2)
Supplement: Additional file 6: Figure S3. — miRNAs and isomiRs classified to the miR-23 (A) and miR-148 (B) families, harboring the same seed sequences. Reference/canonical miRNA found in miRBase, corresponding to specific pre-miRNA was marked in bold and underlined. Underlined sequence of miR-23a/b isomiR could not be uniquely assigned to one member of given family. (PDF 149 kb) [file 12864_2015_2172_MOESM6_ESM.pdf]

A

Reference miR-23a

Pre-miR-23a CGGCUGGGGUUCCUGGGGAUGGGAUUUGCUGCCUGUCACAA**AUCACAUUGCCAGGGAUUUCC**AAUCGACC

	Norm. reads	
AUCACAUUGCCAGGGAUUUCCAAU	4.1	
AUCACAUUGCCAGGGAUUUCCAA	14.2	
AUCACAUUGCCAGGGAUUUCCA	116.0	dominant isomiR
AUCACAUUGCCAGGGAUUUCC	288.1	reference miRNA
AUCACAUUGCCAGGGAUUUC	29.4	
AUCACAUUGCCAGGGAUUU	6.5	
AUCACAUUGCCAGGGAUU	8.1	

Reference miR-23b

Pre-miR-23b CUCUGGCUGCUUGGGUUCCUGGCAUGCUGAUUUGUGACUUAAGAUUAAA**AUCACAUUGCCAGGGAUUACCA**CGCAGCCAC

	Norm. reads	
AUCACAUUGCCAGGGAUUACCAC	44.1	
AUCACAUUGCCAGGGAUUACCA	127.8	reference miRNA
AUCACAUUGCCAGGGAUUACC	332.0	dominant isomiR
AUCACAUUGCCAGGGAUUAC	18.4	
AUCACAUUGCCAGGGAUU	8.1	

Seed sequence (2-8 nt)	Norm. reads
UCACAUU	988.7

B

Reference miR-148a-5p / Reference miR-148a-3p

Pre-miR-148a GAGGC**AAAGUUCUGAGACACUCCGACU**CUGAAUAUGAUAGAAG**UCAGUGCACUACAGAACUUUGU**CUC

	5p sequence	Norm. reads	3p sequence	Norm. reads	
reference miRNA	AAAGUUCUGAGACACUCCGACU	13.6	UCAGUGCACUACAGAACUUUGUCU	17.5	
	AAAGUUCUGAGACACUCCGA	3.4	UCAGUGCACUACAGAACUUUGUC	145.7	
			UCAGUGCACUACAGAACUUUGU	39038.3	reference miRNA
			UCAGUGCACUACAGAACUUUG	749.6	dominant isomiR
			UCAGUGCACUACAGAACUUU	91.2	
			UCAGUGCACUACAGAACUU	16.4	
			UCAGUGCACUACAGAACU	6.4	
			CAGUGCACUACAGAACUUUGU	4.1	
			AGUGCACUACAGAACUUUGU	646.1	
			AGUGCACUACAGAACUUUG	12.6	

Reference miR-148b-5p / Reference miR-148b-3p

Pre-miR-148b AUUUGAGGU**GAAGUUCUGUUAUACACUCAGGC**UGUGGCUCUCUGAAAG**UCAGUGCAUCACAGAACUUUGU**CUCGAAAGCU

	5p sequence	Norm. reads	3p sequence	Norm. reads	
reference miRNA	GAAGUUCUGUUAUACACUCAGGC	4.2	UCAGUGCAUCACAGAACUUUGUCU	7.4	
	GAAGUUCUGUUAUACACUCAGG	4.6	UCAGUGCAUCACAGAACUUUGUC	147.1	
			UCAGUGCAUCACAGAACUUUGU	11230.9	reference miRNA
			UCAGUGCAUCACAGAACUUUG	405.4	dominant isomiR
			UCAGUGCAUCACAGAACUUU	7.9	
			AGUGCAUCACAGAACUUUGU	204.7	
			AGUGCAUCACAGAACUUUG	7.5	

Seed sequence (2-8 nt)	Norm. reads
AAGUUCU	25.8
CAGUGCA	51857.4
AGUGCAC	4.1
GUGCACU	870.9
