# Supplementary material for: Expression of microRNAs and isomiRs in the porcine endometrium: implications for gene regulation at the maternal-conceptus interface
Source: BMC Genomics. 2015 Nov 6;16:906. doi: 10.1186/s12864-015-2172-2 (PMC4636777; doi:10.1186/s12864-015-2172-2)
Supplement: Additional file 10: Figure S4. — Cross-species comparison of 3’UTRs of putative target genes of miR-140-3p and isomiR miR-140-3p 5’ DEL U. (PDF 95 kb) [file 12864_2015_2172_MOESM10_ESM.pdf]

PUTATIVE TARGETS miR-140-3p(seed ACCACAG)

ACVR2B 3'UTR

```
      CAGGCACCAAGAUGG---GACACCAU    miR    (8mer)
             ||||        |||||||
GCAGUGGAUCUUUGUUCCUUUUUACUGUGGUAAUUUUAGAAAUGAGUG              hsa
GCAGUGGAUCUUUGUUCCUUUUUACUGUGGUAAUUUUAGAAAUGAGUG              ptr
GCAGUGGAUCUUUGUUCCUUUUUACUGUGGUAAUUUUAGAAAUGAGUG              mml
GC-GUGGCUCUUUGCUCCUUUUUCCUGUGGUCAUUUUAGAAACGAGUG              mmu
UGCGUGAAUCCUCGCUCCUUUUUCCUGUGGUAAUUUUAG-AAACGAGU              rno
GCAGUGGAUCUUUGUUCCUUUUUACUGUGGUAAUUUUAGAAAUGAGUG              cfa
GCAGUGGAUCUUUGUUCCUUUUUACUGUGGUAAUUUUAGAAAUGAGUG              eca
GCAGUGGAUCUUUGUUC-UUUUUACUGUGGUAAUUUUAGAAAUGAGUG              bta
GCAGUGGAUCUUUGUUCCUUUUUACUAUAGUAAUUUUAGAAAUGAGUG        ssc

CAGGCACCAAGAUGGGACACCAU       miR    (8mer/7mer-A1)
               |||||||
UGGUUUGAUUUUGUGCUGUGGUA-UUUUUUUUCCCUUAGAAUAAUUUU              hsa
UGGUUUGAUUUUGUGCUGUGGUA-UUUUUCUUCCCUUAGAAUAAUUUU              ptr
UGGUUUGAUUUUGUGCUGUGGUA-UUUUUCUUCCCUUGGAAUAAUUUU              mml
--AUCUGAUUUUGUGCUGUGGUA--UUUUCUUGUCUCAGAAUACUUUC              mmu
--------UUUUGUGCUGUGGUAUUUUUUUUUGUUUCAGACUACUUUC              rno
----AUUAUUUUGUGCUGUGGUA-UUUUUCUUCCCUCGGAAUAAUUUU              cfa
----ACUAUUUUGUGCUGUGGUA-UUUUUCUUCCCUUGGAAUAAUUUU              eca
UAAAACUAUUUUGUGCUGUGGUAUUUUUUCCUCCCUCGGAAUGAUUUU              bta
UAAAGU-AUUUUGUGGUGUGGUA-UUUUUCUUCCCUCAGAAUAAUUUU              ssc
```

PUTATIVE TARGETS isomiR mir-140-3p (seed CCACAGG)

LPAR2 3’UTR

```
           CAGGCACCAAGAUGGGACACCA isomiR (8mer/7mer-A1)
                         |||||||
UCA--CUGCGU-AUCCUUGGUAAGCCCUGUGGACUGGUUCCUG          hsa
UCA--CUGCGU-AUCCUUGGUAAGCCCUGUGGACUGGUUCCUG          ptr
UCA--CUGUGU-AUCCUUGGUAAGCCCUGUGGACUG-UUCCUG          mml
UUA--CUGUGUUAUCUCUGG--AUGUCUGUGGACUGCUGCUUC          mmu
UUA--CUGUAC-AUCUCUGGC-AUGUCUGUGGACUGCUGCUUC          rno
UUA--CCACUC-AUCCCUGGUAAACCCUGUGGACUACUUCCCU          cfa
UUA--CUGCAU-AUCCCUGGUAAACUCUGUGGACUGCUAUCUC          eca
UUUUUCUGCAC-AUGCCUGGUAACCCCUGUGGACUGCUUUCUU          bta
UUA--CCACAC-AUCCCUGGUAAACUCUGUGGACUGCUUCCUU          ssc
```

LIF 3’UTR

```
CAGGCACCAAGAUGGGACACCA isomiR (7mer-m8)
              |||||||
UUUGAUGAAUGUACCCUGUGGGGAUGUUUCAUACUGACAGAUU          hsa
UUUGAUGAAUGUACCCUGUGGGGAUGUUUCAUACUGACAGAUU          ptr
UU-GAUGAAUGUACCCUGUGGGGAUGUUUCAUACUGACAGAUU          mml
UUUGAUGAAUGUACCCUGUGGGAAUGUUUCAUACUGACAGAUU          mmu
UUUGAUGAAUGUACCCUGUGGGAAUGUUUCAUACUGACAGAUU          rno
UUUGAUGAAUGUACCCUGUGGGAAUGUUUCAUACUGACAGAUU          cfa
UUUGAUGAAUGUACCCUGUGGGAAUGUUUCAUACUGACAGAUU          eca
UUUGAUGAAUGUACCCUGUGGGAAUGUUUCAUACUGACAGAUU          bta
UUUGAUGAAUGUACCCUGUGGGAAUGUUUCAUACUGACAGGUU          ssc
```

COMMON PUTATIVE TARGETS miR-140-3p (seed ACCACAG) and isomiR miR-140-3p(seed CCACAGG)

KCNMA1 3'UTR

```
               CAGGCACCAAGAUGGGACACCAU miR (7mer-m8/6mer)
                              |||||||
GUAGGCAUUGUCCUGCAGUGAUUUGUCGGACUGUGGUUACUCUG         hsa
GUAGGCAUUGUCCUGCAGUGACUUGUCAGACUGUGGUUACUCUG         ptr
GUAGGCAUUGUCCUGCAGUGACUUGUCGGACUGUGGUUACUCUG         mml
GUAGGCGUUGUCCUGCAGUGACUUGUCAGACUGUGGUUACUCUG         mmu
GUAGGCAUUGUCCUGCAGUGACCUGUCGGACUGUGGUUACUCUG         rno
GUAGGCGCCGUCCUGAAGUGACUUGUCAGACUGUGGUUACCCUG         cfa
AUAAGCGCCAUCCUAAAGUGACUUGUCAGACUGUGGCUACCGUG         eca
GUAGGCGUCGUCCUGAAGUGACUUGUCAGACUGUGGUUACCCUG         bta
GUAGGUGCUGUCCUGAAGUGACUUGUCAGACCGUGGUUACCCUG         ssc

CAGGCACCAAGAUGGGACACCAU miR (8mer)
               |||||||
CUUUGGGCCAUGA-CCUGUGGUACCUAGUUAUGGUAGUGCAAAU         hsa
CUUUGGGCCAUGA-UCUGUGGUACCUAGUUAUGGUAGUGCAAAU         ptr
CUUUGGACCAUGA-CCUGUGGUACUUAGUUAUGGUAGUGCAAAU         mml
CUUUAGGCCAUGAUUCUGUGGUACCUAGUUAUGAUAGUACAAAU         mmu
CUUUAGGCCAUGACUCAGUGGU-CCUAGUUAUGAUAGUAUAAAU         rno
CUUUAGGCCAUGACCCUGUGGCACUUAGU-AUAUUAGUGCAAAU         cfa
CUUUAUGCCAUGACCCUGUGGUACCUAGU-AUGUUAGUGCAAAU         eca
-----GGCCAUGACCCCAUGGUA-CAUAGUAUGUUACUGCAAAU         bta
CAUUAGGCCAUGACCCUGUGGUACCUAGU-AUGUUAGUGCAAAU         ssc

      CAGGCACCAAGAUGGGACACCA  isomiR      (7mer-m8)
                    |||||||
UUAACUCUUUGGGCCAUGA-CCUGUGGUACCUAGUUAUGGUAGU         hsa
UUAACUCUUUGGGCCAUGA-UCUGUGGUACCUAGUUAUGGUAGU         ptr
UUAACUCUUUGGACCAUGA-CCUGUGGUACUUAGUUAUGGUAGU         mml
UGAACUCUUUAGGCCAUGAUUCUGUGGUACCUAGUUAUGAUAGU         mmu
UGAACUCUUUAGGCCAUGACUCAGUGGU-CCUAGUUAUGAUAGU         rno
UUAAUUCUUUAGGCCAUGACCCUGUGGCACUUAGU-AUAUUAGU         cfa
UUAAUUCUUUAUGCCAUGACCCUGUGGUACCUAGU-AUGUUAGU         eca
--------------------------------------------         bta
CUAUUCCAUUAGGCCAUGACCCUGUGGUACCUAGU-AUGUUAGU         ssc
```

SIRT1 3’UTR

```
  CAGGCACCAAGAUGGGACACCAU     miR   (8mer)
                 |||||||
UGUUUAAAUACCUAUCACUGUGGUAGAGCUUGCAUUGA-UCUUUUCCAC          hsa
UGUUUAAAUACCUAUCACUGUGGUAGAGCUUGCAUUGA-UCUUUUCCAC          ptr
--UA-AAUAC-CUAUCACUGUGGUAGAGCUUGCAUUGA-UCUUUUCCAC          mml
UAAUUAAAUACCUAUCACUGUGGUAGAGCCUGCAUAGA-UCUUCACCAC          mmu
UAAUUAAAUACCUAUCACUGUGGUAGAGCCUCCAUAGA-UCUUCACCAC          rno
UGUUUAAAUACCUAUCACUGUGGUAGAGCUUGCAUUGCUUCUUUCCCAC          cfa
UGUUUAAAUACCUGUCACUGUGGUAGAGCAUGCACUGAUCCUUUCCCAC          eca
UGUUUAAACACCUAUCACUGUGGUAGAGCUUGCAUUGA-UCCUUUCCAC          bta
UGUUUAAAUACCUAUCACUGUGGUAGAGCUUGCAUUGAUCCUUUUCCAC          scc


               CAGGCACCAAGAUGGGACACCA     isomiR      (7mer-m8)
                             |||||||
CGUUCAAAUGAAGAUGG-CUUUUGUACUUCCUGUGGACAUGUAGCAAUG          hsa
CGUUCAAAUGAAGAUGG-CUUUUGUACUUCCUGUGGACAUGUAGUAAUG          ptr
UGUUCAAAUGAAGAUGG-CUUUUGUACUUCCUGUGGACAUGUAGUAAUG          mml
UGC----------AUCG-UUUUUGUACUUGCUGCAGAC-GUG--G-UAA          mmu
UGC----------AUGGUUUUUUGUACUUGCUGCAGAC-GUG--G-UAA          rno
UGU-----UGAAGAUGG-CUUUUGUACUUCCUGUGGACAUGUAGUAAUG          cfa
UGUUCAAAUGAAGAUGG-CUUUUGUACUUCCUGUGGACAUGUAGUAAUG          eca
CGUUCAAAUGAAGAUCG-CUUUUGUACUUCCUGUGGACAUGUAGUAAUG          bta
UGUUCAAAUGAAGAUGG-CUUUUGUACUUCCUGUGGACAUGUGGUAAUG          ssc
```
