# Supplementary material for: Expression of microRNAs and isomiRs in the porcine endometrium: implications for gene regulation at the maternal-conceptus interface
Source: BMC Genomics. 2015 Nov 6;16:906. doi: 10.1186/s12864-015-2172-2 (PMC4636777; doi:10.1186/s12864-015-2172-2)
Supplement: Additional file 11: Table S7. — Primers, GenBank accession numbers and product lengths of miRNA synthesis/transport-related genes studied in real-time RT-PCR analysis. (DOCX 17 kb) [file 12864_2015_2172_MOESM11_ESM.docx]

**Additional file 11: Table S7**. Primers, GenBank accession numbers and product lengths of miRNA synthesis/transport-related genes studied in real-time RT-PCR analysis.

| Gene name | Primer sequences | GenBank accession number | Product length (bp) |
| --- | --- | --- | --- |
| *DROSHA*  *DICER*  *AGO1*  *AGO2*  *AGO3*  *AGO4*  *TNRC6A*  *TARBP2*  *DGCR8*  *XPO5*  *PPIB^#^*  *GAPDH^#^*  *ACTB^#^*  *HPRT^#^*  *RNA18S* ^#^ | F: 5’ TTACCAACCCTGGGACGAAAC 3’  R: 5’ AACTCAACTGTGCAGGGCGTAT 3’  F: 5’ CAATCAACACGGCCATTGGA 3’  R: 5’ AACAATGGAGGCTCGAAGAGGT 3’  F: 5’ ATTGATGTCTCAGCCACTGCCT 3’  R: 5’ CTTGATCTCCTTGGTGAAGCGTAC 3’  F: 5’ TTACAAGTCGGACAGGAGCAGA 3’  R: 5’ AGTCGCTCTGATCATGGTTGAG 3’  F: 5’ TACACCGCCAATCCACTTCCT 3’  R: 5’ GCCAACTCACCCGAGAGACA 3’  F: 5’ CATCAGTCTGTGAGACCTGCCAT 3’  R: 5’ TTGACACGCTGGGAGTCTGTTAG 3’  F: 5’ GCCTCCAGCACAACCTCTCA 3’  R: 5’ GGATTCAGGCCACCGTTGT 3’  F: 5’ GCAGTCTGAGTGCAACCCTGTT 3’  R: 5’ CCACTCGGCAGGTCATGGTA 3’  F: 5’ CCTCCTCGTAGACCCGAACTG 3’  R: 5’ GCTCTCGGTAAAGCTCACGCTA 3’  F: 5’ATATGCTCTCCGCTGCACAGAC 3’  R: 5’ AATCTGGACCCAGCAATGCA 3’  F: 5’ TTGTGGCCTTGGCTACAGGA 3’  R: 5’ GTTCTCGTCGGGAAAGCGTT 3’  F: 5’ TCGGAGTGAACGGATTTG 3’  R: 5’ CCTGGAAGATGGTGATGG 3’  F: 5’ ACATCAAGGAGAAGCTCTGCTACG 3’  R: 5’ GAGGGGCGATGATCTTGATCTTCA 3’  F: 5’ CTTGGTCAAGCAGCATAATCCA 3’  R: 5’ AGCCTACCACAAACTTGTCTGGA 3’  F: 5’ ACGTCTGCCCTATCAACTTTCG 3’  R: 5’ CTTGGATGTGGTAGCCGTTTCT 3’ | NM_001100412.1  NM_030621.3  NM_001194976  NM_001164623  NM_024852  NM_017629  NM_014494.2  XM_003355410.2  NM_001206910.1  XR_135433.1  XM_001927047.2  AF017079.1  U07786  NM_001032376.2  NR_046261.1 | 126  164  141  121  124  164  126  134  130  129  155  219  366  128  118 |

^#^ Reference gene.
