# Supplementary material for: Expression of microRNAs and isomiRs in the porcine endometrium: implications for gene regulation at the maternal-conceptus interface
Source: BMC Genomics. 2015 Nov 6;16:906. doi: 10.1186/s12864-015-2172-2 (PMC4636777; doi:10.1186/s12864-015-2172-2)
Supplement: Additional file 14: Table S8. — miRNA/isomiR names, sequences and assays ID of molecules validated by stem-loop real-time RT-PCR in the porcine endometrium. (DOCX 18 kb) [file 12864_2015_2172_MOESM14_ESM.docx]

| miRNA/isomiR | Sequence | Assay ID |
| --- | --- | --- |
| *miR-1* | UGGAAUGUAAAGAAGUAUGUA | 000385 |
| *miR-23b* | AUCACAUUGCCAGGGAUUACCA | 008376_mat |
| *miR-191-5p* | CAACGGAAUCCCAAAAGCAGCUG | 002299 |
| *miR-205* | UCCUUCAUUCCACCGGAGUCUG | 000509 |
| *miR-203*^§^ | GUGAAAUGUUUAGGACCACUAG | 000507 |
| *miR-302*^§^ | UAAACGUGGAUGUACUUGCUUU | 000530 |
| *miR-23a DEL C^$^* | AUCACAUUGCCAGGGAUUUC | 008219_mat |
| *miR-30a-3p DEL C^$^* | CUUUCAGUCGGAUGUUUGCAG | 244137_mat |
| *miR-191-5p DEL G^$^* | CAACGGAAUCCCAAAAGCAGCU | 000490 |
| *let 7d-3p^#^* | AGAGGUAGUAGGUUGCAUAGUU | 002283 |
| *miR-148a^#^* | UCAGUGCACUACAGAACUUUGU | 000470 |
| *miR-199b-5p^#^* | CCCAGUGUUUAGACUAUCUGUU | 462091_mat |

**Additional file 14: Table S8.** miRNAs/isomiRs names, sequences and assay IDs of molecules validated by stem-loop real-time RT-PCR in the porcine endometrium.

^§^ miRNA not known in pig

^$^ isomiRs

^#^ Reference miRNA
