# Supplementary material for: Expression of microRNAs and isomiRs in the porcine endometrium: implications for gene regulation at the maternal-conceptus interface
Source: BMC Genomics. 2015 Nov 6;16:906. doi: 10.1186/s12864-015-2172-2 (PMC4636777; doi:10.1186/s12864-015-2172-2)
Supplement: Additional file 15: Table S9. — Names, Assay IDs, GenBank accession numbers and product length of selected genes analyzed in real-time PCR in an in vitro study. (DOCX 15 kb) [file 12864_2015_2172_MOESM15_ESM.docx]

**Additional file 15: Table S9.** Names, Assay IDs, GenBank accession numbers and product length of selected genes analyzed in real-time PCR in an *in vitro* study.

| Gene name | Assay IDs | Gene Bank accession number | Product length (bp) |
| --- | --- | --- | --- |
| *ACVR2B* | Ss03391780_m1 | NM_001005350.1 | 89 |
| *KCNMA1* | Ss03394390_m1 | NM_214219.1 | 119 |
| *LIF* | Ss03391458_m1 | NM_214402.2 | 104 |
| *LPAR2* | Ss03377227_u1 | NM_001162401.1 | 53 |
| *SIRT1* | Ss03374091_m1 | NM_001145750.1 | 67 |
| *ACTB^#^* | Ss03376081_u1 | AK237086.1 | 77 |
| *HPRT1^#^* | Ss03388274_m1 | NM_001032376.2 | 73 |

^#^ Reference gene.
